# Identification of novel MITEs (miniature inverted-repeat transposable elements) in *Coxiella burnetii*: implications for protein and small RNA evolution

**DOI:** 10.1186/s12864-018-4608-y

**Published:** 2018-04-11

**Authors:** Shaun Wachter, Rahul Raghavan, Jenny Wachter, Michael F. Minnick

**Affiliations:** 1 Program in Cellular, Molecular and Microbial Biology, Division of Biological Sciences, University of Montana, Missoula, MT USA; 2 Biology Department and Center for Life in Extreme Environments, Portland State University, Portland, OR USA; 3 Laboratory of Zoonotic Pathogens, Rocky Mountain Laboratories, National Institute of Allergy and Infectious Diseases, National Institutes of Health, 903 South 4th St, Hamilton, MT USA

**Keywords:** Q fever, Transposon, MITE, Genetic element, *Coxiella burnetii*, Evolution, Small non-coding RNA

## Abstract

**Background:**

*Coxiella burnetii* is a Gram-negative gammaproteobacterium and zoonotic agent of Q fever. *C. burnetii*’s genome contains an abundance of pseudogenes and numerous selfish genetic elements. MITEs (miniature inverted-repeat transposable elements) are non-autonomous transposons that occur in all domains of life and are thought to be insertion sequences (ISs) that have lost their transposase function. Like most transposable elements (TEs), MITEs are thought to play an active role in evolution by altering gene function and expression through insertion and deletion activities. However, information regarding bacterial MITEs is limited.

**Results:**

We describe two MITE families discovered during research on small non-coding RNAs (sRNAs) of *C. burnetii*. Two sRNAs, Cbsr3 and Cbsr13, were found to originate from a novel MITE family, termed QMITE1. Another sRNA, CbsR16, was found to originate from a separate and novel MITE family, termed QMITE2. Members of each family occur ~ 50 times within the strains evaluated. QMITE1 is a typical MITE of 300-400 bp with short (2-3 nt) direct repeats (DRs) of variable sequence and is often found overlapping annotated open reading frames (ORFs). Additionally, QMITE1 elements possess sigma-70 promoters and are transcriptionally active at several loci, potentially influencing expression of nearby genes. QMITE2 is smaller (150-190 bps), but has longer (7-11 nt) DRs of variable sequences and is mainly found in the 3′ untranslated region of annotated ORFs and intergenic regions. QMITE2 contains a GTAG repetitive extragenic palindrome (REP) that serves as a target for IS1111 TE insertion. Both QMITE1 and QMITE2 display inter-strain linkage and sequence conservation, suggesting that they are adaptive and existed before divergence of *C. burnetii* strains.

**Conclusions:**

We have discovered two novel MITE families of *C. burnetii*. Our finding that MITEs serve as a source for sRNAs is novel. QMITE2 has a unique structure and occurs in large or small versions with unique DRs that display linkage and sequence conservation between strains, allowing for tracking of genomic rearrangements. QMITE1 and QMITE2 copies are hypothesized to influence expression of neighboring genes involved in DNA repair and virulence through transcriptional interference and ribonuclease processing.

**Electronic supplementary material:**

The online version of this article (10.1186/s12864-018-4608-y) contains supplementary material, which is available to authorized users.

## Background

*C. burnetii* is a Gram-negative, obligate intracellular gammaproteobacterium and the etiologic agent of Q fever in humans. Q fever is an acute, flu-like illness that can present with pneumonitis, hepatitis and malaise. In less than 5% of cases, chronic infection can develop with potentially life-threatening endocarditis as the most common manifestation [1]. *C. burnetii* undergoes a biphasic life cycle in which it alternates between a metabolically-active, replicative large-cell variant (LCV) and a dormant, spore-like small-cell variant (SCV) [2]. Upon inhalation of SCV’s by a mammalian host, alveolar macrophages internalize the bacteria and trap them within a highly acidic (pH ~ 4.5) parasitophorous vacuole that has features of a mature phagolysosome [3]. *C. burnetii* has adapted to survive in this acidic environment, where it forms a replicative niche. Dot/Icm effectors are translocated to the host cell in a type IV secretion system-dependent manner in order to establish and maintain the vacuole [4]. Lipopolysaccharide is another critical virulence determinant in *C. burnetii* [5], although it has been found to be truncated (rough) in some strains, including the Nine Mile phase II laboratory strain, RSA 439 [6]. Interestingly, the Dugway 5 J108-111 strain has a full-length lipopolysaccharide, but is avirulent [7]. Dugway is considered to be the most primitive of the sequenced *C. burnetii* strains based on a larger genome with apparently less reductive evolution than virulent strains, such as RSA 493 [8]. It is hypothesized that Dugway either contains a gene(s) that impedes infection in humans, or that the virulent RSA 493 strain has some altered virulence gene(s) rendering it infective [9].

*C. burnetii*’s genome suggests that it is a relatively recent obligate intracellular pathogen, based upon the high number of pseudogenes and selfish genetic elements [10]. Among these elements are an intein [11], two group I introns [12], an intervening sequence (IVS) [13], and TEs, including multiple copies of IS1111 [14]. The IS1111 transposon has been studied extensively and found to preferentially insert into a palindromic DNA sequence that is widely distributed throughout the *C. burnetii* genome [15]. This palindromic DNA sequence has been described as a GTAG repetitive extragenic palindrome (REP), although the nature and distribution of the REP has not been described [16]. There is little information on other families of TEs in the *C. burnetii* genome.

MITEs are non-autonomous class II TEs with defective or missing transposase genes. As such, they can only be mobilized in *trans* by transposases from related transposons [17]. Most bacterial MITEs consist of 4-30 bp terminal inverted repeats (TIRs) with a TA dinucleotide at their termini. MITEs are typically small (100-400 bp) and do not encode proteins; rather, their transcripts generate highly stable stem-loop structures [18]. MITE insertions have been implicated in virulence by fostering a plastic genome that enhances acquisition of virulence traits [19] and through physical insertions that alter ORFs and directly lead to virulence phenotypes [20]. Promoter regions and ORFs are common features of bacterial MITEs [21–24]. Moreover, integration host factor (IHF)-binding sites and methyltransferse binding domains have been reported [22, 25]. While most MITEs integrate into intergenic regions of the genome, some have been reported: a) in structural RNA genes [26], b) in protein-encoding genes to create in-frame protein fusions [27], and c) proximal to genes whose transcripts are regulated by the corresponding MITE RNA [28, 29]. Thus, MITEs can potentially interact at DNA, RNA or protein levels in a host bacterium, depending upon their structure and genomic sites of integration. *C. burnetii* was recently shown to produce at least 15 small non-coding RNAs (sRNAs) [30]. In this report, we show that *C. burnetii*’s sRNA 3 (Cbsr3), CbsR13, and a newly defined sRNA, Cbsr16, arose from two novel MITE families of the pathogen. Furthermore, we demonstrate how these novel MITE families can serve as a timeline for IS1111 transposition based upon their linkage and sequence conservation between strains. Finally, we show that although MITE copies show linkage and sequence conservation, an indel in a potential virulence-associated gene (*enhC*) affected by QMITE2 has created a truncated version of the gene in the virulent RSA 493 strain as compared to the avirulent Dugway strain.

## Methods

Discontiguous MegaBLAST (https://blast.ncbi.nlm.nih.gov/Blast.cgi) was used as a local alignment program using default parameters to identify regions of homology to Cbsr13 and Cbsr16 in the *C. burnetii* genome (strains RSA 493; GenBank accession number AE016828.3 and Dugway 5 J108-111; GenBank accession number CP000733.1). In order to compare the various QMITE loci in the RSA 493 genome, multiple sequence alignments of QMITE copies were performed using MUSCLE alignments via Geneious version 11.0.2 software with the default settings [31] (https://www.geneious.com/download/). Phylogenetic analyses of various groups of QMITE insertions were carried out by first trimming the MUSCLE alignments utilizing Gblocks version 0.91b software [32] (http://molevol.cmima.csic.es/castresana/Gblocks.html). This served to eliminate poorly aligned and highly divergent regions in the various alignments. The default parameters are exceptionally stringent and are catered towards longer input sequences. Thus, the minimum block length was reduced to four, and gap positions were allowed for half of the input sequences at each aligned position in order to accommodate the relatively shorter input sequences. Phylogenetic trees of these trimmed alignments were then constructed using FastTree version 2.1 [33] (http://www.microbesonline.org/fasttree/#FAQ). The generalized time-reversible model of nucleotide evolution was used and phylogeny was inferred using maximum likelihood. The resulting Newick tree file was visualized using FigTree version 1.4.3 (http://tree.bio.ed.ac.uk/software/figtree/). To support our designations of QMITEs as MITEs, supplemental MITE predictions of the *C. burnetii* RSA 493 genome were performed using MUSTv2 software [34] (http://www.healthinformaticslab.org/supp/resources.php). Predicted RNA secondary structures used to confirm the presence of TIRs were generated using mfold [35] (http://unafold.rna.albany.edu/?q=mfold). In order to demonstrate the potential for transcription of QMITE inserts, prediction of sigma-70 consensus promoter elements and Rho factor-independent terminators in QMITE inserts was performed using BPROM (http://www.softberry.com/berry.phtml?topic=bprom&group =programs&subgroup=gfindb) and ARNold (http://rna.igmors.u-psud.fr/toolbox/arnold/), respectively. CIRCOS software [36] (http://circos.ca/software/ download/ circos/) was used to visualize and depict positions of QMITEs on the *C. burnetii* chromosome. RNA-Seq data [Sequence Read Archive (SRA) database under accession number SRP041556] were analyzed using a custom pipeline, although various nesoni version 0.128 applications for processing high-throughput sequence data were also used (http://www.vicbioinformatics.com/software.nesoni.shtml). Transcripts per million (TPM) were calculated using custom perl and python scripts that can be accessed through GitHub (https://github.com/shawachter/TPM_Scripts). The Artemis genome browser was used to visualize alignment files generated from ambiguous and unambiguous read data (http://www.sanger.ac.uk/science/tools/artemis) [37]. Other figures were created using Powerpoint 2010 software (Microsoft, Redmond, WA).

## Results

### CbsR3 and CbsR13 loci are members of a novel MITE family

**Fig. 1:**
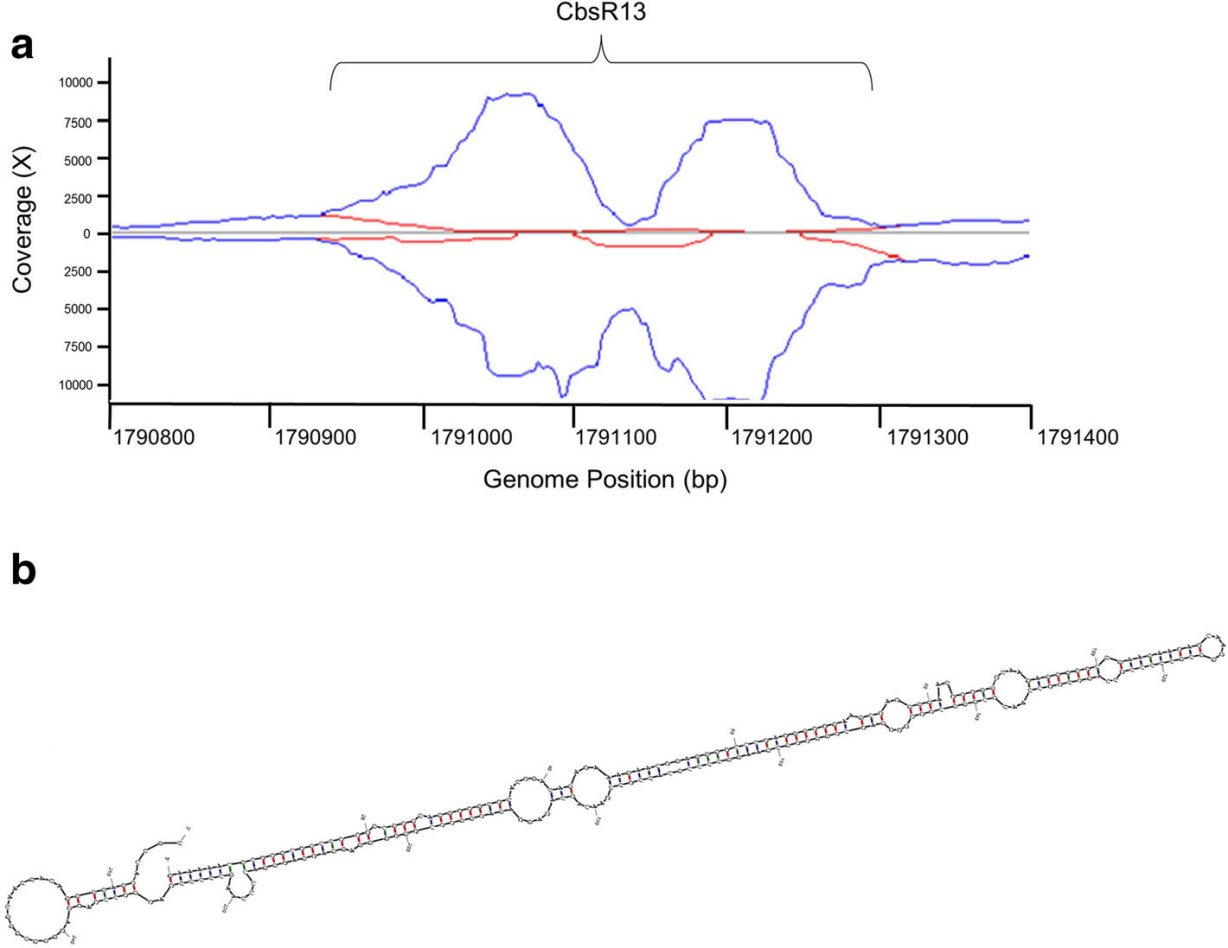
Ambiguous and unambiguous reads map to the CbsR13 locus. **a** Artemis view of reads mapping to the CbsR13 locus (RSA 439 genome). The x-axis indicates the location (bp) on the chromosome and the y-axis indicates coverage of reads mapping to that location. Reads above the y-axis indicate antisense reads, whereas reads below the y-axis indicate sense reads mapping to that genomic location. Blue lines signify ambiguous reads mapping to this locus, while the red lines denote unambiguous reads. **b** mfold secondary structure prediction of the CbsR13 sRNA. Red, blue, and green lines forming stem structures indicate G-C, A-U, and G-U base-pairing, respectively (predicted ∆G = − 128.5 kcal/mol)

**Fig. 2:**
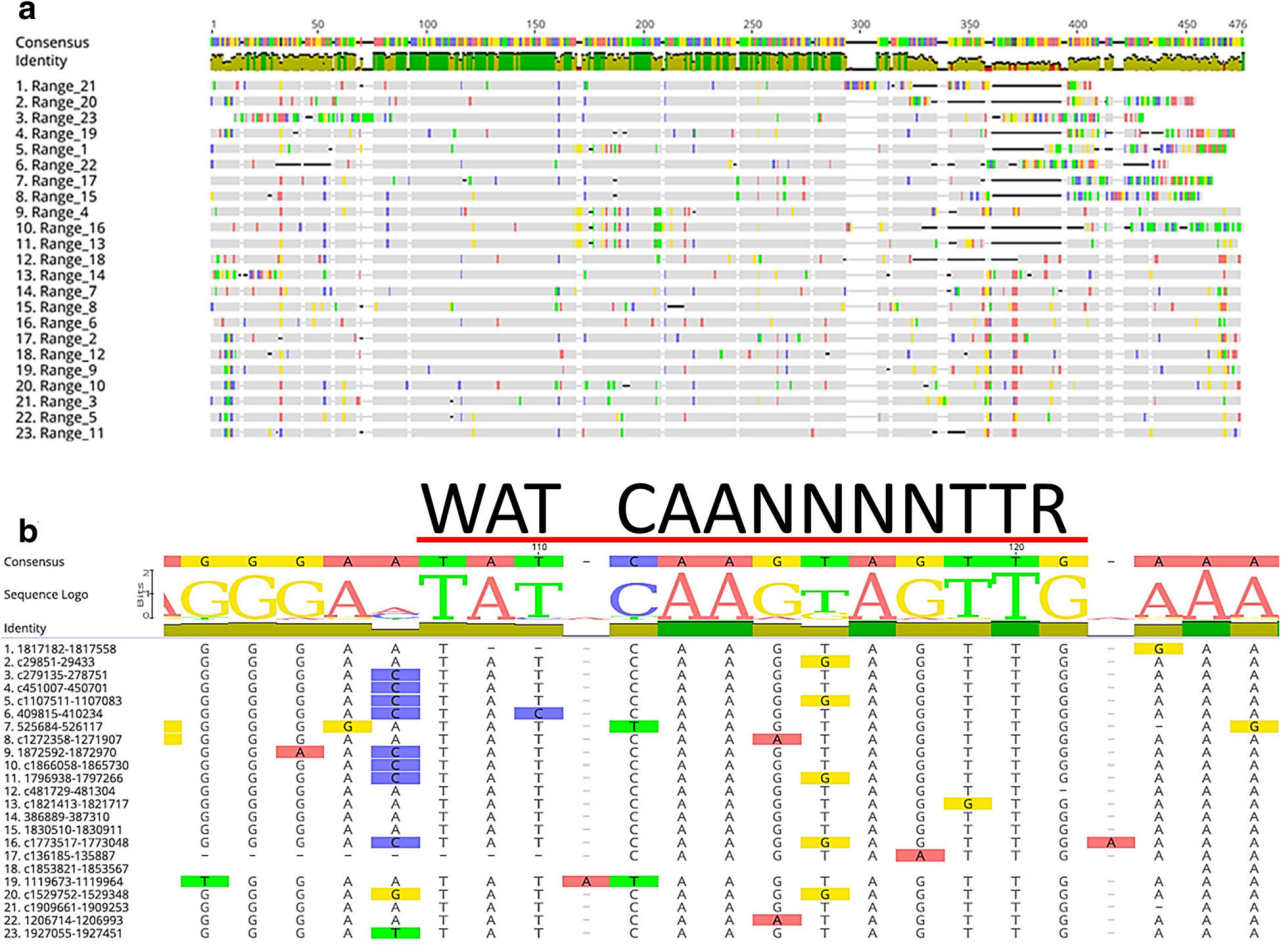
CbsR13 loci contain a canonical IHF-binding site. **a** MUSCLE sequence alignment of discontiguous megaBLAST hits (> 75% of input sequence) associated with the extended-CbsR13 input sequence. Conserved bases appear as gray blocks, while unaligned bases appear as green, yellow, blue, and red bands, corresponding to T, G, C, and A bases, respectively. An identity indicator is shown above the sequence alignment, where height signifies conservation of bases at that position, with a color indicator for overall identity between aligned ranges (green: 100%, yellow: 20-99%, red: 0-19%). The consensus sequence is shown above the identity indicator as colored bands indicating bases as described above. **b** The same alignment as shown in (A), focusing on the potential IHF-binding site. The sequence above the red line indicates the consensus IHF-binding site utilizing nucleotide notation, and above the alignment is a sequence logo where the height of the displayed bases indicates the relative identity of the aligned base at that position

Cbsr13 was originally identified as a *C. burnetii* sRNA by RNA-Seq analysis of the transcriptome [30]. It is often helpful to analyze both ambiguous and unambiguous reads associated with any RNA-Seq data. Ambiguous reads refer to those reads that can’t be aligned to one specific area of the genome because multiple copies of that sequence exist in the genome. Unambiguous reads refer to those that could only be mapped to one region of the genome. Upon visualization of ambiguous and unambiguous reads that map to the CbsR13 locus, we discovered that there were many ambiguous reads associated with it (Fig. 1a). We also found that CbsR13 RNA produced a stable predicted secondary structure resembling a very long palindromic sequence (Fig. 1b). Although a megaBLAST search produced several hits of high homology, the divergent nature of the CbsR13 sequences necessitated use of a discontiguous megaBLAST search, which identified dozens of sequences with significant homology to CbsR13 in the genome. Specifically, the search identified 44 ranges, with E values of 8e-11 to 3e-123. Of these hits, 21 were at least 75% of the length of CbsR13 (> 232 bp). It was noted upon alignment of the regions flanking these sequences that some of the ranges contained truncated 5′ ends and elongated 3′ ends. An artificial sequence combining the native CbsR13 sequence and the 3′ extension (see Additional file 1) was thus used as an input for another discontiguous megaBLAST search. This search revealed 45 ranges, with E values from 9e-10 to 5e-123. Twenty-three of these hits were at least 75% of the input sequence length (> 350 bp). A multiple alignment and phylogenetic analysis of these 23 sequences is shown in Fig. 2a and Additional file 2, respectively. The remaining 22 elements ranged in size from 39 to 321 bp (not shown), possibly representing degenerate forms of the original nucleotide sequences. One megaBLAST hit for the extended-CbsR13 corresponded to a large portion of the CbsR3 gene sequence (i.e., nt 481,609-481,806) (see Fig. 2a, range 2) [30]. This result suggests that the two sRNAs share a common ancestor, although unambiguous TPM values from RNA-Seq show that CbsR13 is expressed at a markedly higher level relative to CbsR3 (Additional file 3). Confirming what is seen in Fig. 1a, the ambiguous TPMs associated with CbsR3 and CbsR13 are much higher than the unambiguous TPMs, indicating that additional CbsR13 loci are transcriptionally active (Additional file 3). Indeed, a sigma-70 promoter search using BPROM predicts a promoter in the forward strand and two promoters in the reverse strand of the input sequence (Additional file 1).

**Fig. 3:**
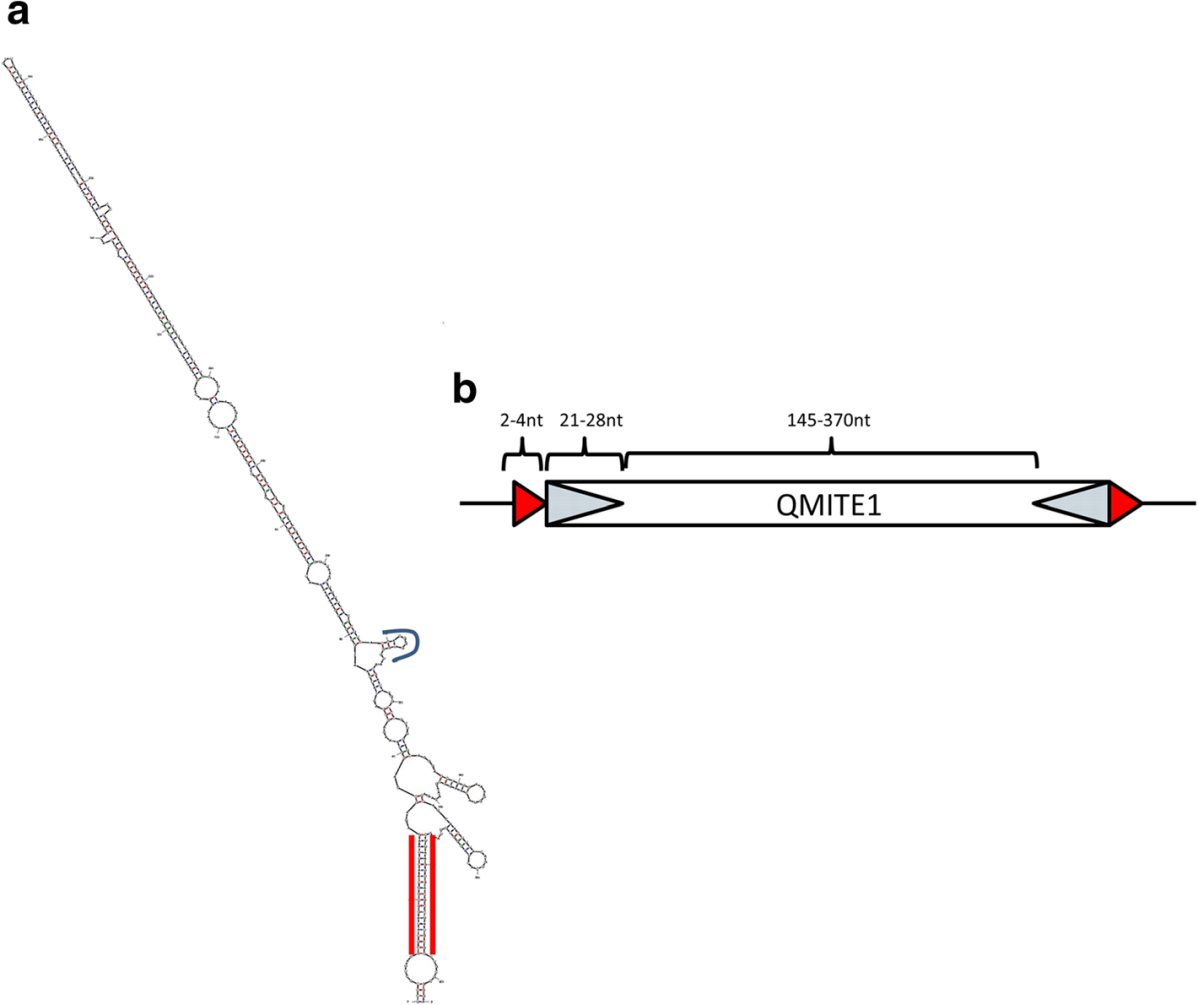
CbsR13 loci represent a novel MITE, called QMITE1. **a** mfold secondary structure prediction of a selected QMITE1 (range 5; predicted ∆G = − 192.72 kcal/mol). Red bars bracket the TIRs and the blue line indicates the location of the potential IHF-binding site. **b** Model of QMITE1 depicting DRs as red arrow heads and the TIRs as hatched arrow heads. Length ranges for these features are also shown

A common motif associated with bacterial TEs is an IHF-binding site [23]. IHF is a bacterial DNA-binding protein that binds to a specific DNA motif and facilitates bending of the DNA. It is thought that this bending aids in transposition of the locus [38]. The consensus IHF-binding nucleotide sequence is WATCAANNNNTTR [39]. Although IHF-binding sites are common in bacterial TEs, they are not always present in MITEs [23]. A manual search through the aligned ranges in Fig. 2a, though, led to the discovery of a well-conserved IHF-binding site (Fig. 2b). We chose Range 5 (Fig. 2a) as a representative for this repeated sequence due to its completeness, and utilized mfold to visualize where this IHF-binding site was located and to see if the sequence had a TIR that could aid in the element’s categorization as a MITE. As shown in Fig. 3a, it is clear that the element has a TIR of 21 bp in length. Based on the length of the element (~ 400 bp), the TIR, and the multiple loci scattered throughout the *C. burnetii* RSA 493 genome, we conclude that this element is a bona fide MITE. Moreover, no similar MITEs have been previously described, and BLASTn searches found no orthologues in other genomes. Thus, we can conclude that this is a novel MITE that we designate as QMITE1. Other ranges in Fig. 2a generated similar predicted secondary structures, with corresponding TIRs ranging from 21 to 28 nts (not shown). MUSTv2 software was also employed to confirm QMITE1 as a MITE (Additional file 4) [32]. Using stringent parameters, MUSTv2 identified eight of the top ten most homologous ranges to the extended-CbsR13 input sequence and also identified 2-4 bp DRs of nucleotide compositions WW, SS, or GAAG. From this information, a model of QMITE1 was generated and is shown in Fig. 3b.

### QMITE1 copies encode basic peptides and overlap with annotated genes

Along with being transcriptionally active, 19 QMITE1 copies fully contain short, annotated ORFs that encode predicted peptides with an average isoelectric point (pI) of 12.4. These basic peptides can be divided into three major groups based on sequence similarity (Additional file 5), and they constitute the entire DUF1658 family of small, uncharacterized *C. burnetii* proteins in the Pfam database [40].

Other annotated genes that are affected by QMITE1 insertions mainly encode hypothetical proteins of unknown function. However, QMITE1 copies also overlap with several functional genes, including: *ubiB* C-terminal 2-bp overlap, *pntAA* C-terminal 42-bp overlap, *mutT* C-terminal 26-bp overlap, CBU_2058 proline/betaine transporter C-terminal 49-bp overlap, *nagZ* C-terminal 50-bp overlap, and CBU_2020 glutamate transporter C-terminal 3-bp overlap. The effect of these QMITE1 insertions in the 3′ untranslated regions of these genes could not be determined, although other MITE insertions in 3′ untranslated regions have been observed to translationally repress the affected genes [41].

### The CbsR16 locus is a member of a second novel MITE family

**Fig. 4:**
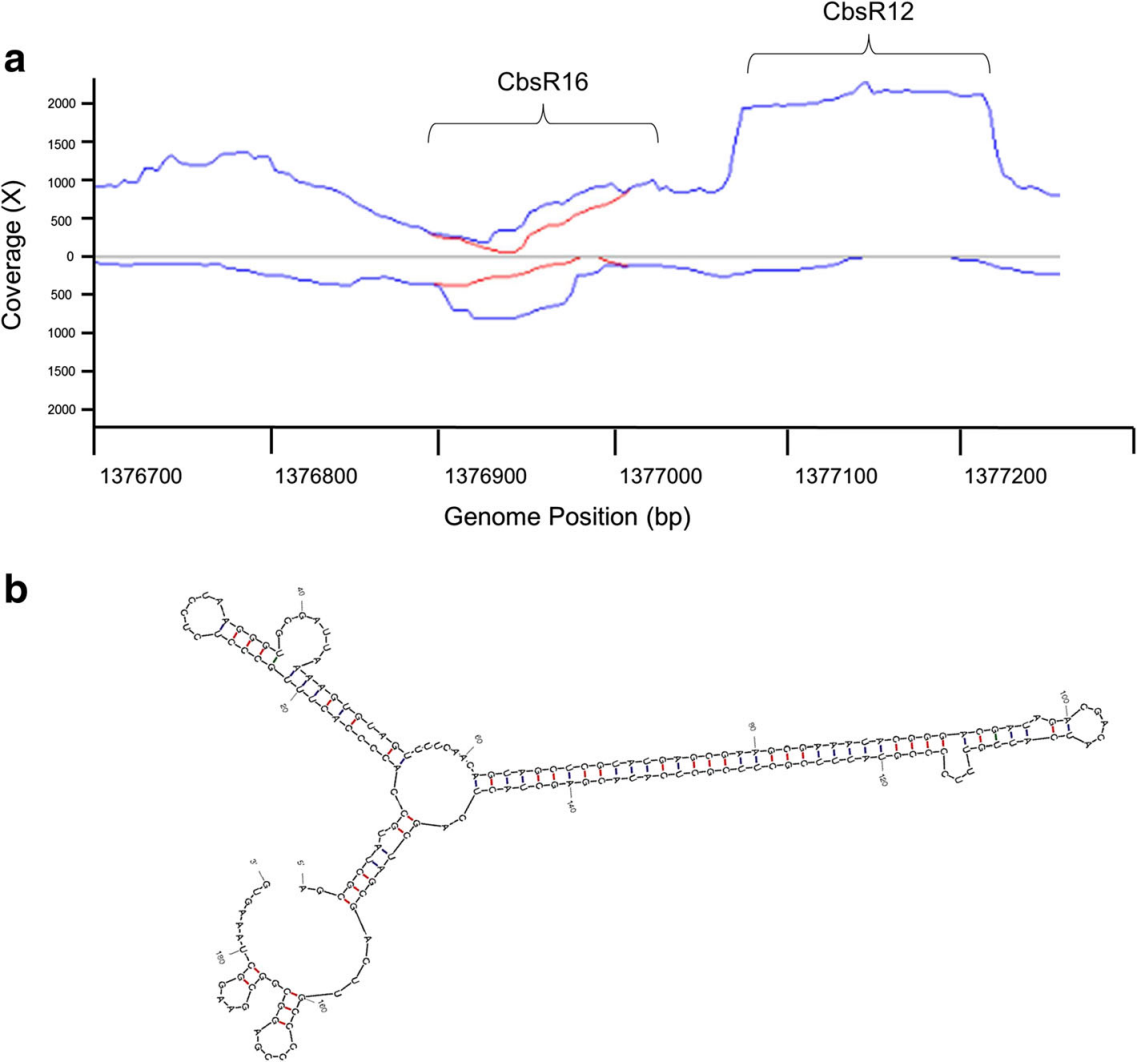
CbsR16 is lowly transcribed, with some ambiguous reads mapping to it. **a** Artemis view of reads mapping to the CbsR16 locus (RSA 439 genome). The x-axis shows the location (bp) on the chromosome and the y-axis indicates coverage of reads mapping to that location. Reads above the y-axis indicate antisense reads, whereas reads below the y-axis indicate sense reads mapping to that genomic location. Blue lines signify ambiguous reads mapping to this locus, while red lines signify unambiguous reads. **b** mFold prediction of the CbsR16 sRNA secondary structure (∆G = − 85.24 kcal/mol). Red, blue, and green lines forming stem structures indicate G-C, A-U, and G-U base-pairing, respectively

We recently identified a new sRNA termed Cbsr16 while analyzing Cbsr12; a sRNA that is significantly upregulated during *C. burnetii*’s intracellular infection of host cells [30]. The Cbsr16 gene is located immediately downstream of the CbsR12 gene, which shares a bi-directional Rho-independent terminator with Cbsr16 (data not shown). When viewing the CbsR16 locus with the Artemis genome browser, it was clear that there was minor differential expression of the locus when taking ambiguous reads into consideration (Fig. 4a). Additionally, when we analyzed CbsR16 using mfold, the predicted secondary structure was highly stable (Fig. 4b). Moreover, although QMITE1 is significantly transcribed at more than one location in the *C. burnetii* genome, CbsR16 is transcribed at a considerably lower level (Additional file 3), with very minor TPM differences between mapped unambiguous and ambiguous transcripts. This indicates that although other sequences homologous to CbsR16 may exist in the RSA 493 genome, only the locus adjacent to CbsR12 is transcribed to any significant level. The strong secondary structure and minor presence of ambiguously mapped reads of CbsR16, though, warranted a genome-wide search for similar sequences.

**Fig. 5:**
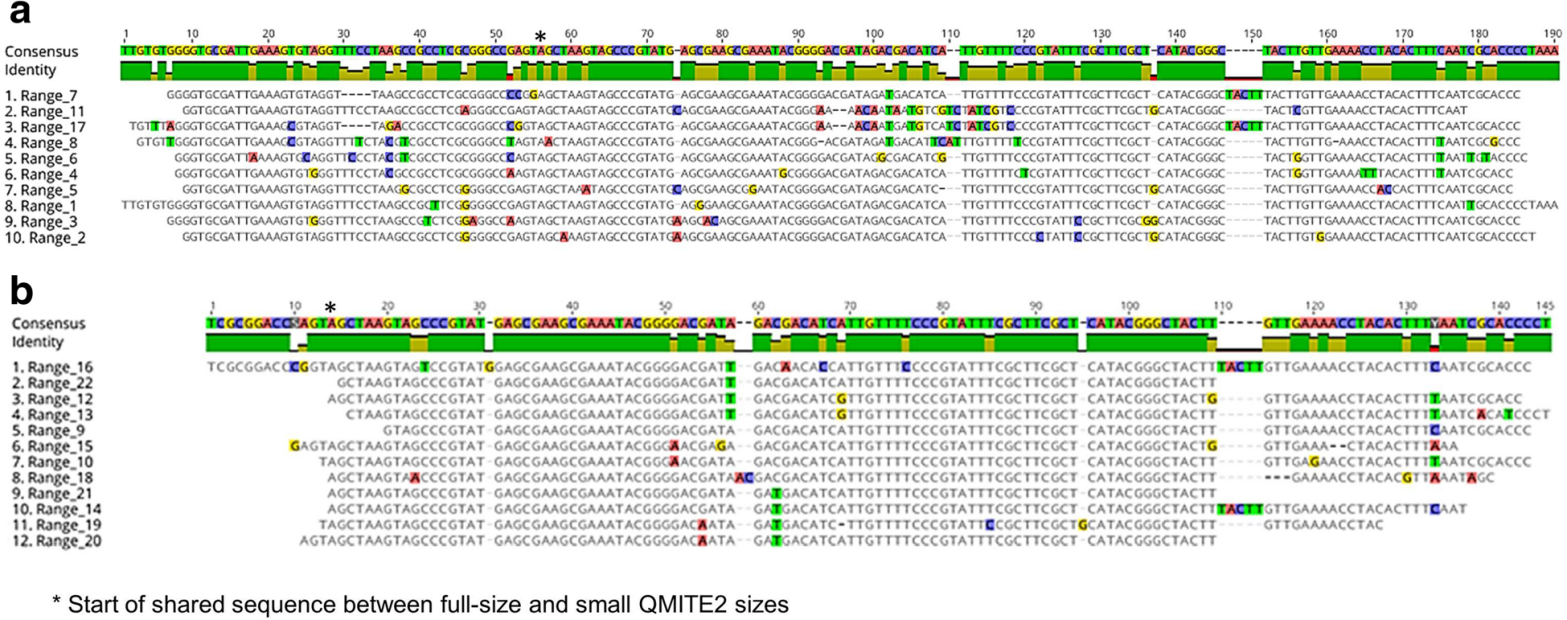
CbsR16 loci have full-size and small versions. **a** MUSCLE sequence alignment of discontiguous megaBLAST hits that returned full-size versions of the CbsR16 locus. Conserved bases appear as gray blocks, while unaligned bases appear as green, yellow, blue, and red bands, corresponding to T, G, C, and A bases, respectively. An identity indictor is shown above the sequence alignment, where the height signifies conservation of bases at that position, with a color indicator for overall identity between aligned ranges (green: 100%, yellow: 20-99%, red: 0-19%). Above this identity indicator is the consensus sequence, appearing as colored bands indicating bases as described above. **b** As in (A), except the MUSCLE alignment displays the top discontiguous megaBLAST hits (> 75% of input sequence) associated with the CbsR16 locus, excluding all full-size hits. An asterisk indicates equivalent positions in the full-size and small versions of QMITE2

**Fig. 6:**
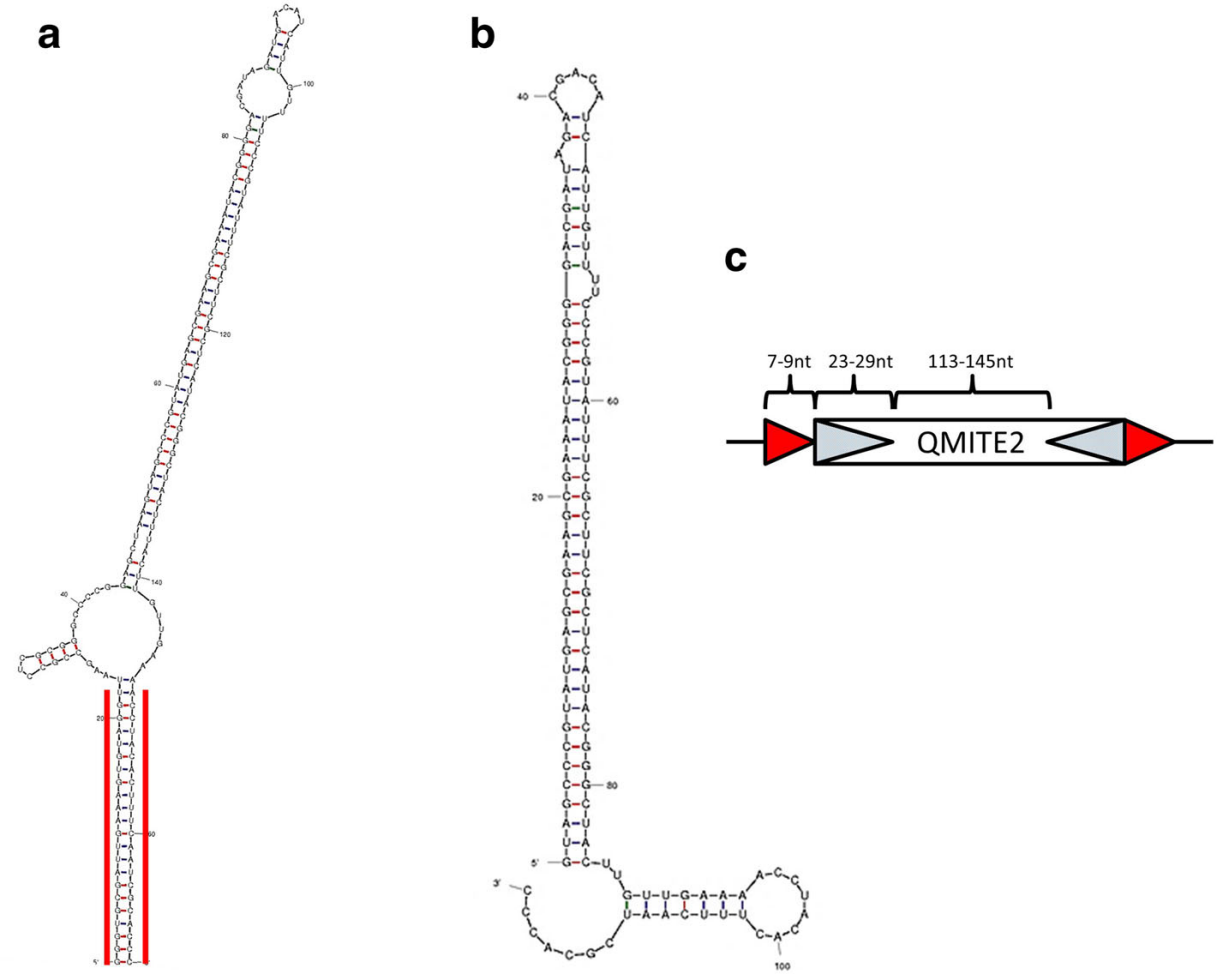
CbsR16 loci comprise another novel MITE family, termed QMITE2. **a** mFold prediction of the RNA secondary structure of a full-size version of the CbsR16 repeated locus (range 7; predicted ∆G = − 113.09 kcal/mol). Red, blue, and green lines forming stem structures indicate G-C, A-U, and G-U base-pairing, respectively. Red lines bracket the identified TIR. **b** As in (A), but depicting the secondary structure prediction of a small version of the CbsR16 repeated locus (range 9; predicted ∆G = − 67.7 kcal/mol). (**C**) Model of QMITE2 depicting DRs as red arrow heads and TIRs as hatched arrow heads. Length ranges for these features are also shown

A discontiguous megaBLAST search with Cbsr16 resulted in 78 hits with E-values ranging from 1e-07 to 4e-33. We initially divided these 78 hits into two groups: full-size sequences and smaller sequences. From these pools, we aligned those that covered at least 75% of the input CbsR16 sequence. The full-size versions (Fig. 5a) have a 5′ sequence of ~ 40 nts that is apparently missing in smaller versions of the element (Fig. 5b). Phylogenetic trees for these full-size and smaller versions were constructed and can be seen in Additional file 6 and Additional file 7, respectively. As with QMITE1, we generated representative predicted secondary structures for the full-size (Range 7, Fig. 6a) and small ranges (Range 9, Fig. 6b). Although there are no IHF-binding sites in the CbsR16-like sequences, the full-size ranges have TIRs and are flanked by unique DRs of 7-9 bp, while the smaller ranges are essentially REP elements. Interestingly, these REP elements were previously reported in *C. burnetii*, although their status as a truncated MITE was not recognized [16]. Taken as a whole, the size (~ 190 bp), presence of TIRs and DRs, and their distribution across the RSA 493 genome suggest that the CbsR16-like loci are indeed MITEs. We therefore propose to designate this family of elements as QMITE2. A model of QMITE2 is shown in Fig. 6c. The smaller QMITE2 copies strongly resemble a REP element; i.e., they do not contain TIRs nor do they have discernible DRs in flanking genomic regions.

As observed with QMITE1, QMITE2 copies may also affect certain annotated ORFs. Although they do not encode annotated genes like some QMITE1 copies, there is some overlap with neighboring functional genes, including a C-terminal 1-bp overlap with *kdgK*, a C-terminal 8-bp overlap with *ogt*, a C-terminal 7-bp overlap with *recN*, a C-terminal 10-bp overlap with CBU_2078 Fic-Family protein, and a C-terminal 6-bp overlap with *ruvB*. Additionally, although MUSTv2 identified QMITE1 in the RSA 493 genome, it was unable to find QMITE2 under stringent parameters. However, a full-size QMITE2 copy was identified using less stringent parameters (data not shown). The inability for MUSTv2 to identify QMITE2 most likely reflects the filtering parameters of the program itself. Namely, the program searches for copies of the MITE with similar DR’s. If a copy with a similar DR is not found, it will filter it out. QMITE2 has unique DR’s for each copy, making it difficult to detect.

### QMITE2 loci are hot-spots for IS1111 insertion

While parsing various QMITE2 ranges, we found that 20 of the 21 annotated IS1111 TEs in the RSA 493 genome possessed a small QMITE2 located ~ 400 bp downstream of their stop codons. These small QMITE2 ranges were aligned and shown in Additional file 8. A phylogenetic tree of these transposon-associated QMITE2 insertions was created and is shown in Additional file 9. These ranges are nearly identical to the other small QMITE2 ranges (Fig. 5b), except that they are missing 10-20 bp at the 5′ end. Upon closer inspection, these “missing” bases are actually located 5′ of the IS1111, indicating that the transposon inserted into this region of QMITE2. Indeed, this has been described before, although the insertion site was not previously recognized as a MITE [15]. It is worth noting that these QMITE2 copies are more divergent than their IS1111-free counterparts, implying neutral selection while they are associated with IS1111. Interestingly, of the twenty IS1111 insertions in QMITE2 copies, only one clearly inserted into a full-length QMITE2 locus, as the TIR is still discernible up- and down-stream of the transposon insertion. The other QMITE2 loci may also have been full-length once, but their flanking sequences presumably diverged rapidly after insertion.

### QMITE2 is not specific to *C. burnetii*

**Fig. 7:**
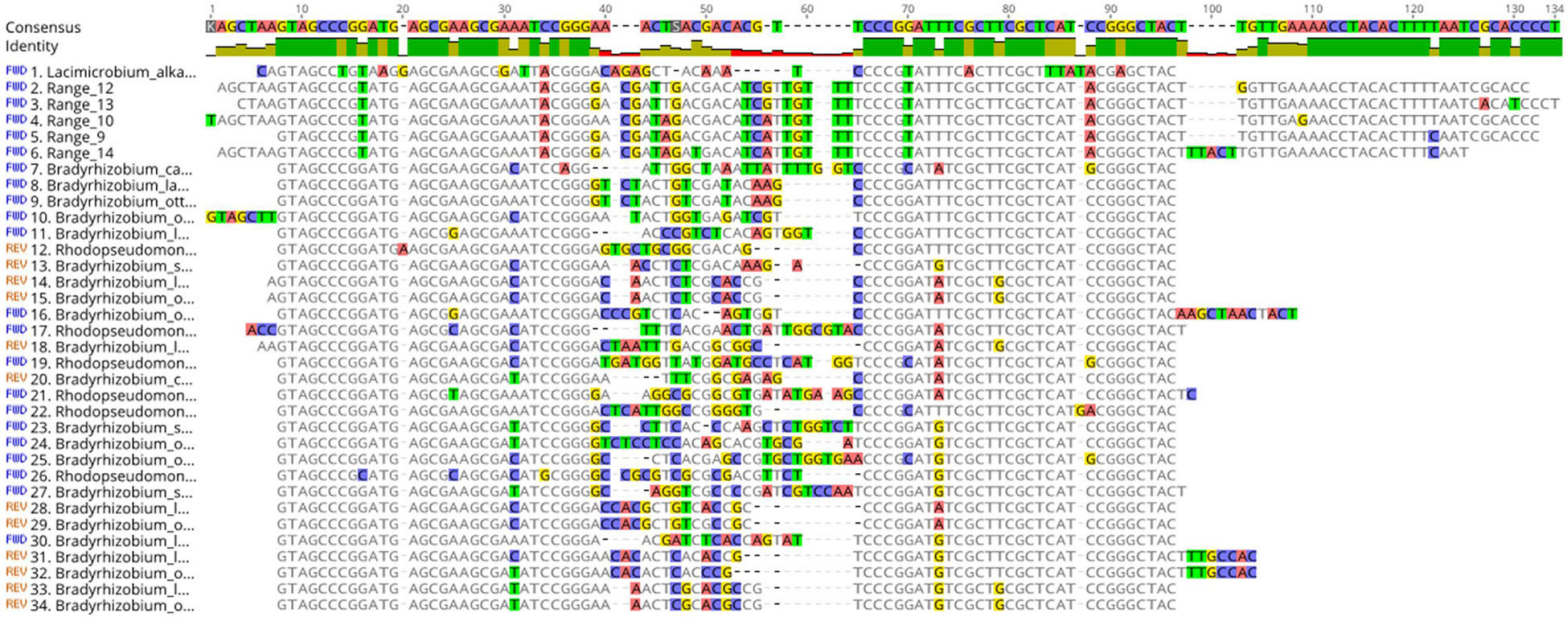
QMITE2 is not unique to *C. burnetii*. MUSCLE sequence alignment of discontiguous megaBLAST hits that returned QMITE2 sequences in other organisms. Conserved bases appear as gray blocks, while unaligned bases appear as green, yellow, blue, and red bands, corresponding to T, G, C, and A bases, respectively. An identity indicator is shown above the sequence alignment, where the height signifies conservation of bases at that position with a color indicator for overall identity between aligned ranges (green: 100%, yellow: 20-99%, red: 0-19%). Above this identity indicator is the consensus sequence, appearing as colored bands indicating bases as described above. Ranges 12, 13, 10, 9, 14 in the sequence alignment refer to small QMITE2 ranges included in the BLAST as shown in Fig. 5b

Unlike QMITE1, QMITE2 is apparently not unique to *C. burnetii*. A discontiguous megaBLAST search using the CbsR16 sequence yielded hits in multiple alphaproteobacteria, including *Bradyrhizobium* spp*.* and *Rhodobacter* spp. These hits had sizes of 83-100 nucleotides in length with E-values ranging from 1E-04 to 8E-07. QMITE2 also appeared in one location in *Lacimicrobium alkaliphilum*, a gammaproteobacteria. These sequences were aligned to the small version of QMITE2 (Fig. 7) and a phylogenetic tree was constructed (Additional file 10**)**. The alignment indicates that although the majority of the sequence corresponding to the predicted stem structure of the small QMITE2 is conserved, the palindromic “tip” (see Fig. 6b, bases 31-53) is more divergent among the alphaproteobacteria shown in the alignment. These results suggest that a majority of the palindromic stem structure may serve some function in *Bradyrhizobium* and *Rhodopseudomonas* spp., while the entirety of this stem is under purifying selection in *C. burnetii*. It’s also worth noting that the 3′ portion of QMITE2 is missing from the alphaproteobacterial MITEs. The 3′ end of QMITE2 comprises half of the TIR formed in the full-length QMITE2 suggesting that full-length QMITE2 never existed in the alphaproteobacterial species or was present further back in their evolutionary histories.

### Full-length QMITE2 displays inter-strain linkage and sequence conservation in *Coxiella*

Due to the unique DRs produced by individual full-length QMITE2 insertions, we were interested to see if these DRs displayed inter-strain linkage conservation. To accomplish this, full-length QMITE2 ranges were found in the *C. burnetii* Dugway strain and the DRs produced by these inserts were compared to those produced by QMITE2 inserts in the RSA 493 strain. If there were two DRs that were identical in sequence between strains, we determined if the associated QMITE2 copies were linked by observing syntenic genome blocks that were produced via genome rearrangements as the strains diverged [7]. We discovered that the Dugway strain contains 12 full-length QMITE2 copies versus 10 in RSA 493 (Table 1). Furthermore, seven of the nine discernible DRs in the RSA 493 strain had perfect homologs in the Dugway strain and displayed perfect linkage and sequence conservation. The single unique DR in RSA 493 without a counterpart in Dugway resulted from an IS1111 insertion in the corresponding position in Dugway’s genome. Likewise, 11 of the 12 full-length QMITE2 copies in Dugway had unique DRs associated with them and seven of these had perfect homologs in RSA 493, two had a IS1111 inserted into the corresponding position in RSA 493, one position belonged to a genomic segment unique to Dugway, and the final position displayed a QMITE2 inversion in RSA 493, leaving no discernible DR (Table 1). In summary, most DRs are conserved in both strains with a few lost via deletion, IS1111 insertion, or genome inversion events.

**Table 1:** Full-size QMITE2 copies exhibit inter-strain linkage conservation

Strain	Range	TIR Length	DR Length	DR Sequence	Homolog?
RSA 493	c1006608-1,006,428	25	^a^	^a^	No^b^
	1,066,751-1,066,922	29	^a^	^a^	^a^
	1,380,514-1,380,685	26	7	TCAGRGG	No^c^
	c1168547-1,168,380	24	9	CCGTCAATA	Yes
	c1360856-1,360,689	23	9	CACATCGAT	Yes
	1,988,089-1,988,258	23	7	CAACATTW	Yes
	1,586,332-1,586,502	23	9	GTTGGCGCG	Yes
	220,015-220,188	25	8	GGGGTGTT	Yes
	c970302-970,140	24	7	GCTACTT	Yes
	1,252,325-1,252,500	24	9	TTCTGTTTA	Yes
Dugway	c334562-334,393	23	9	GTTGGCGCG	Yes
	c1836762-1,836,594	25	8	GGGGTGTT	Yes
	2,151,397-2,151,569	23	8	CAACATTW	Yes
	117,745-117,908	22	^a^	^a^	^a^
	c1299129-1,298,960	23	9	CCGTCAATA	Yes
	c374053-373,882	31	9	AATTTTAAC	No^b^
	1,295,396-1,295,566	26	9	GTATCRTCC	No^c^
	1,561,569-1,561,721	21	13	CCTTCTTCTTTSA	No^d^
	1,384,775-1,384,900	23	9	TTCTGTTTA	Yes
	1,261,463-1,261,626	17	9	GGGCTTTCA	No^c^
	c565652-565,819	25	9	CACATCGAT	Yes
	c1003105-1,002,901	24	7	GCTACTT	Yes

^a^No discernible DR

^b^QMITE2 inversion in other strain

^c^IS1111A insertion in other strain

^d^Genomic segment deleted in other strain

### QMITE1 and QMITE2 copies in the RSA 493 and Dugway genomes

**Fig. 8:**
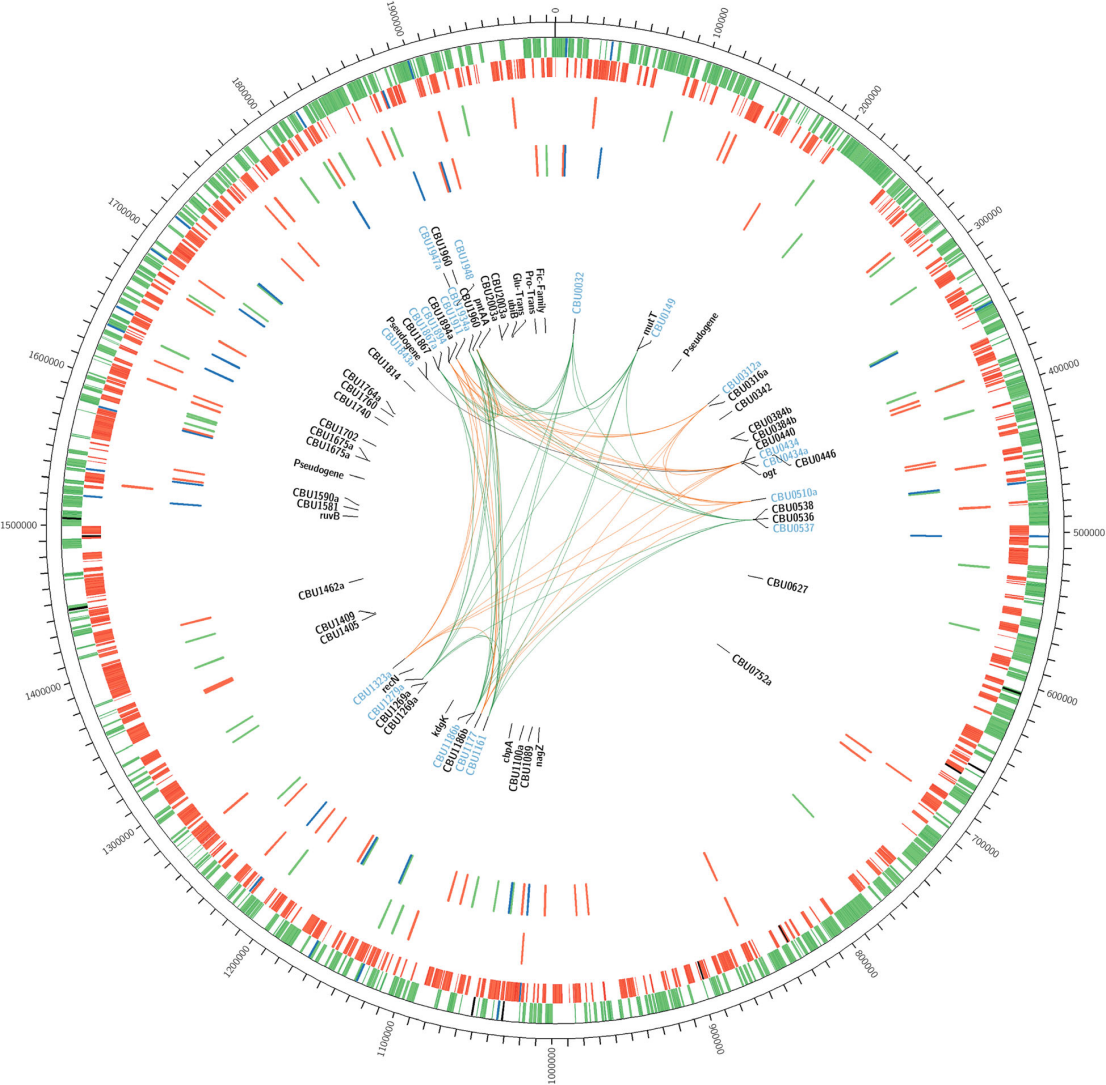
Locations of QMITE1 and QMITE2 insertions in the *C. burnetii* RSA 493 genome. The outer ring depicts the RSA 493 chromosome in 100,000 bp increments. The next ring depicts locations of forward strand ORFs in green, IS1111 locations in blue, and non-IS1111 TEs in black, followed by reverse strand ORFs in red on the next ring also featuring IS1111 in blue and non-IS1111 TEs in black. The next ring depicts all chromosomal QMITE1 locations. Green ticks indicate QMITE1 insertions oriented in the forward, while red ticks indicate QMITE1 insertions in the reverse orientation. The next ring depicts QMITE2 insertions, with green ticks indicating forward insertions, red ticks indicating reverse insertions, and blue ticks indicating IS1111-associated QMITE2 inserts. The following ring labels all of the locus tags for ORFs that have some overlap with either QMITE1 or QMITE2 insertions. ORFs labeled in blue are those that are encoded by QMITE1 insertions and represent the DUF1658 family of proteins. Finally, the colored links between blue-labeled ORFs are indicative of groupings of the proteins coded by these genes (see Additional file 5)

QMITE1 and QMITE2 (full-size and small) copies were mapped against the RSA 493 genome using Circos software (Fig. 8) [36]. We identified 45 copies of QMITE1 and 78 copies of QMITE2 in the RSA 493 genome that in total affect 60 annotated ORFs, with 19 of these ORFs being completely contained within QMITE1 copies and encoding the DUF1658 family of proteins (see Additional file 5). When combined, QMITE1 and QMITE2 copies make up 0.93% of the RSA 493 genome. Interestingly, our analysis revealed that there were generally higher concentrations of QMITE insertions in the second “half” of the genome (~ 1,000,000 – 1,995,488 bp), with small QMITE “deserts”. Accordingly, the first half of the genome was found to contain lower concentrations of QMITEs, with larger deserts (e.g., 570,000 – 690,000 bp) bearing no QMITE inserts.

**Fig. 9:**
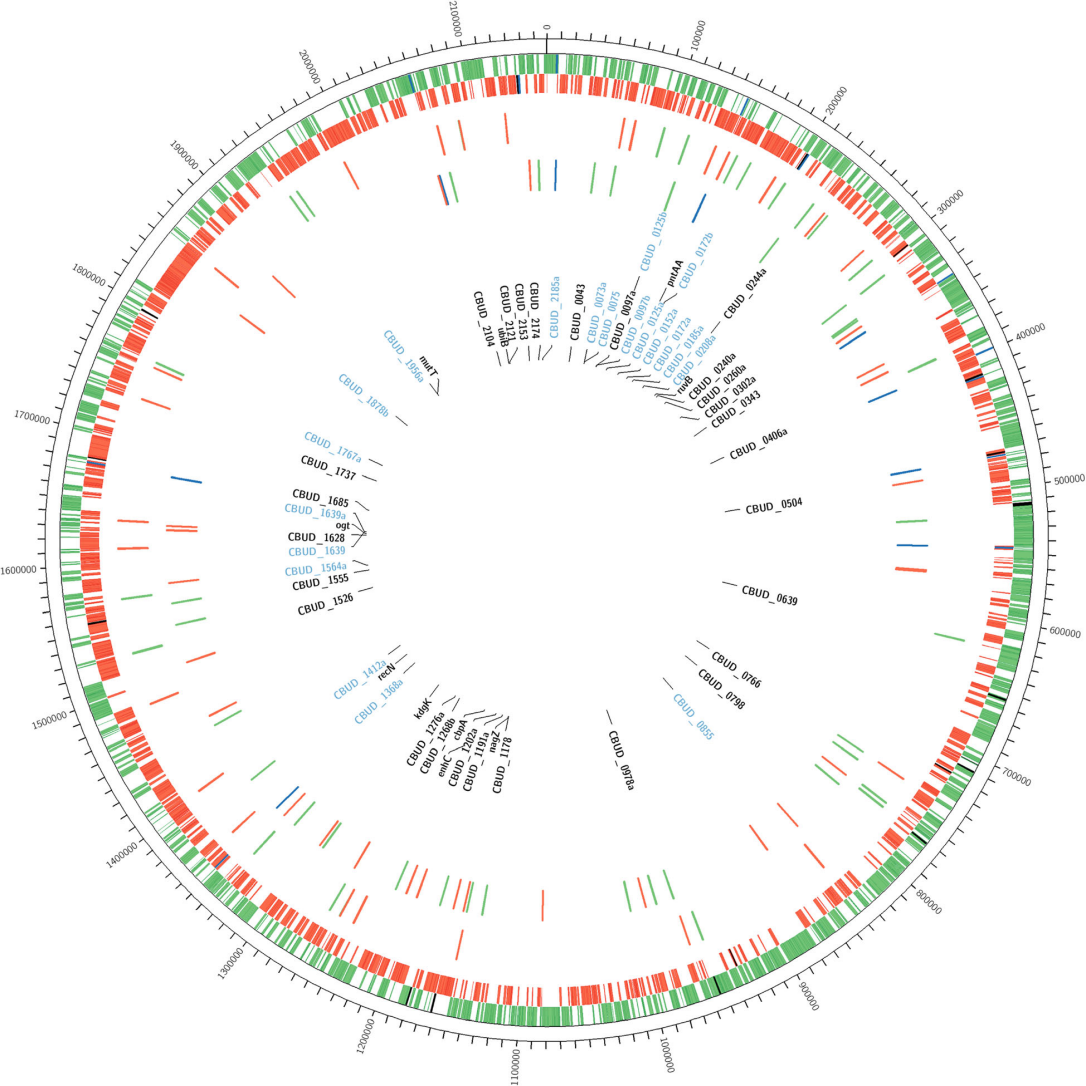
Locations of QMITE1 and QMITE2 insertions in the *C. burnetii* Dugway 5 J108-111 genome. The outer ring depicts the RSA 493 chromosome in 100,000 bp increments. The next ring depicts locations of forward strand ORFs in green, IS1111 locations in blue, and non-IS1111 TEs in black, followed by reverse strand ORFs in red on the next ring also featuring IS1111 in blue and non-IS1111 TEs in black. The next ring depicts all chromosomal QMITE1 locations. Green ticks indicate QMITE1 insertions oriented in the forward, while red ticks indicate QMITE1 insertions in the reverse orientation. The next ring depicts QMITE2 insertions, with green ticks indicating forward insertions, red ticks indicating reverse insertions, and blue ticks indicating IS1111-associated QMITE2 inserts. The following ring labels all of the locus tags for ORFs that have some overlap with either QMITE1 or QMITE2 insertions. ORFs labeled in blue are those that are encoded by QMITE1 insertions and represent the DUF1658 family of proteins. Colored links are omitted because the DUF1658 protein products remain the same between strains and largely depend on how the genome was annotated

The distribution of QMITE1 and QMITE2 in the Dugway genome is displayed in Fig. 9. Due to linkage conservation of QMITE1 and QMITE2 copies between strains, the genomic locations of the QMITE copies are generally the same as RSA 493, although due to divergence between strains, there are some differences in the number of QMITE copies. Specifically, there are 53 copies of QMITE1 and 62 copies of QMITE2 that together comprise 0.91% of the Dugway genome. There are also 56 ORFs affected by MITEs in the Dugway strain. All of the functional annotated genes affected are the same in the two strains, except for the *enhC* gene, which shows a 3′ extension due to an indel linking the gene to a QMITE2 copy. Interestingly, a C-terminally extended EnhC protein has been previously described for the Dugway strain [7].

### QMITE copies affect sRNA genes

New bacterial sRNAs can arise from degraded bacteriophage genes [42]. Similarly, we show that three sRNAs of *C. burnetii* are derived from MITEs. These results suggest that, as shown in eukaryotes [43], genomic parasitic elements can serve as a source for the generation of novel non-coding RNAs of bacteria. For instance, QMITE1 copies have inserted directly downstream of promoter elements for CbsR3 and CbsR13. Moreover, a QMITE2 copy has apparently provided the − 10 promoter element for CbsR16, while the − 35 promoter element is located directly upstream of the QMITE2 insert (Additional file 11). All of these sRNAs show varying levels of expression (see Additional file 3), indicating that they are being actively transcribed. Furthermore, previously published Northern blots have confirmed that CbsR3 and CbsR13 are transcribed and produce sRNA molecules of the expected size [13].

## Discussion

We have described two novel MITE elements in *C. burnetii*, termed QMITE1 and QMITE2. Although their structures and distribution are clear, the nature of their transposition and origin remains indeterminate. Several lines of evidence suggest that QMITE copies are ancient and likely lost the ability to transpose before divergence of present-day *C. burnetii* strains. First, *C. burnetii* RSA 493 contains a plasmid called QpH1 that encodes type 4 secretion system substrates involved in virulence [44]. We could not detect QMITE copies in QpH1, or other *C. burnetii* plasmid types, indicating that either *Coxiella* gained the plasmid after the QMITEs lost the ability to transpose or that the plasmid is too gene-rich to contain stable QMITE copies. Second, the fact that QMITE copies show linkage conservation between strains suggests that they were present before the rearrangement of chromosomes that occurred during divergence of strains. Finally, the presence of QMITE deserts in *C. burnetii* chromosomes (see Figs. 8 and 9), especially between CBU_0664 and CBU_0715, which code for non-IS1111 TEs, implies that horizontal gene transfer (HGT) was involved in forming these regions. Indeed, a recent report has shown that this region (608,000 – 660,000 bp; Fig. 8**)**, is rich in genes that were acquired via HGT, including some LPS biosynthesis genes that are essential to *C. burnetii*’s virulence [45]. The lack of QMITEs in this region indicates that it was acquired after QMITE1 and QMITE2 lost the ability to transpose, but before divergence of strains, since this region displays inter-strain linkage conservation. It is also worth noting that the chromosomal region harboring the *icm/dot* genes involved in type IV secretion display a paucity of QMITE inserts and is flanked by IS1111 TEs that have inserted into QMITE2 copies (see 1,540,000 to 1,580,000 bp in Fig. 8). This suggests that QMITE2 copies indirectly affected the evolution of *C. burnetii* from a free-living to an obligate parasite by fostering genome plasticity.

Interestingly, QMITE insertions can also be used as a marker for the transposition of certain IS1111 TEs. For example, it is likely that the IS1111 transposons at CBU_1217a and CBU_1186 in the RSA 493 strain inserted into these positions after divergence from the Dugway strain, because in Dugway there are full-size QMITE2 copies with discernible DRs that have no IS1111 TEs in these positions. Similarly, the CBUD_0567a IS1111 of Dugway inserted into that position after divergence, since there is a full-size QMITE2 copy at this position in the RSA 493 genome.

The uniqueness of the QMITE1 insert sequence suggests that it may have utility as a molecular signature for detecting *C. burnetii* in clinical or environmental samples. A current detection protocol utilizes PCR to amplify the so-called *htpAB*-repetitive element, which is part of the IS1111 TE [46]. Recent reports, however, have expressed concerns regarding this method due to the existence of IS1111 TEs in *Coxiella*-like endosymbionts, which may confound results obtained from environmental samples [47]. The QMITE1 sequence has variable ends, although it maintains a conserved core across insertions in the *C. burnetii* genome (see Fig. 2a) that could easily serve as a sizeable DNA template for PCR amplification. Also, the abundance of insertion sites in the *C. burnetii* genome should ensure sensitivity of the assay.

Although results suggest that QMITE1 is unique to *C. burnetii*, a relative of QMITE2 was observed in very distantly-related alphaproteobacteria. However, these QMITE2 copies are not full-length and strongly resemble transposon-associated QMITE2 copies (see Additional file 8). These alphaproteobacteria, including several *Bradyrhizobium* and *Rhodopseudomonas* spp., are root nodule-associated, free-living bacteria that also encode several copies of the IS1111 TE in their genomes. There are several possible scenarios that could help explain the occurrence of QMITE2 between these distantly-related organisms. First, *C. burnetii* may have acquired QMITE2 from root nodule-associated bacteria via HGT (or vice versa) during its free-living past. Indeed, *C. burnetii*’s genome contains relics of competence, including an almost-complete type IV pilus system that could have facilitated uptake of foreign DNA [10]. Second, QMITE2 may be ancient, existing long before divergence of alpha- and gamma-proteobacteria. Finally, it is entirely possible that these alphaproteobacteria acquired QMITE2 copies via cut-and-paste transposition of IS1111 following HGT, which in turn left relics of QMITE2 scattered across their respective genomes. This latter explanation is certainly possible since full-size QMITE2 copies are absent in these species and the shorter QMITE2 copies they harbor are highly divergent.

All functional annotated genes affected by QMITE contain insertions located at their 3′ ends. The reason for this preference is unknown but may reflect the general tractability of the C-terminus of proteins to a change in amino acid composition. Indeed, when comparing these protein products to counterparts in *L. pneumophila*, there is no significant difference in the overall masses of the proteins, indicating that QMITE insertions neither extend nor truncate the proteins to a significant degree, although the amino acid composition is altered. These alterations are summarized in Table 2 below. In general, QMITE insertions into these genes increase the hypothetical pI of the encoded protein relative to predicted products lacking the QMITE insert. Such a chimera could have conceivably provided a subtle, adaptive advantage to *C. burnetii* as it transitioned from a free-living bacterium to an obligate intracellular pathogen, as high pI proteins could potentially serve as proton sinks in an acidic host cell phagolysosome. In fact, many *C. burnetii* proteins have been described as having a very high pI, comparable to those found in the human stomach pathogen, *Helicobacter pylori* [10]. This may have been adequate to confer a selective advantage, but the alternative possibility is that QMITE insertions are simply under neutral selection with little to no effect on the fitness of the gene in question. Unfortunately, among the genes listed in Table 2, only orthologues for *recN* and *ruvB* are found in *H. pylori*. Similar to *C. burnetii*, these *H. pylori* (strain 26,695) proteins have a theoretical pI of 5.84 and 5.86, respectively. This suggests that maintenance of an acidic pI was necessary and the minor change caused by the QMITE2 insert in these genes had little effect on fitness. It is also worth noting that there seems to be a preference for QMITE insertions at the 3′ end of DNA-binding genes and genes involved in DNA repair, such as *ogt*, *recN*, *mutT*, and *ruvB*. It is possible that these insertions are simply due to their proximity to these genomic locations during transposon-induced DNA repair. In fact, it has been found that transposition of TEs is increased upon genotoxic stress in bacteria [48]. There also does not appear to be any QMITE elements that affect the 5′ end of genes with known functions. This is most likely due to the necessity for a promoter element upstream; a feature that may not be provided by the QMITE insertion. Alternatively, there may be a transcription factor binding site(s) upstream of the ORF that is necessary for regulation of that gene. In contrast, there seems to be no locational preference for QMITE insertion into annotated hypothetical proteins, wherein QMITE insertions sometimes appear in-frame in the middle of the ORF (e.g., QMITE2 insertions in CBU_0752a and CBU_1269a).

**Table 2:** QMITE effects on functional gene products

Gene	QMITE Type	Overlap length (bp)	Amino acids conferred	pI without insert	pI with insert	Gene function
*ubiB*	1	2	(STOP)	N/A	N/A	Ubiquinone Biosynthesis
CBU_2020	1	3	(STOP)	N/A	N/A	Glutamate antiporter
*pntAA*	1	42	AQTHRRQLKGAR(STOP)	6.93	8.79	Redox, proton transport
*mutT*	1	26	LQQDIITQ(STOP)	5.1	4.96	Mutational DNA repair
CBU_2058	1	49	LVVPAQTHRRQLKGAR(STOP)	9.97	10.15	Proline/Betaine transporter
*nagZ*	1	50	ESQQRLLSFSRFTTGG(STOP)	5.76	5.88	Mureine tripeptide recycling
*kdgK*	2	1	(STOP)	N/A	N/A	Pentose phosphate pathway
*ogt*	2	8	TK(STOP)	7.67	8.32	DNA alkylation repair
CBU_2078	2	10	SAK(STOP)	6.16	6.29	Regulation of cell division
*recN*	2	7	SV(STOP)	6.05	6.05	DNA repair
*ruvB*	2	6	E(STOP)	5.85	5.73	Holliday Junction resolution; DNA repair

An intriguing aspect of QMITE inserts is the influence they can have on sRNAs, depending on where they insert into the genome. It has been suggested that a class of MITEs in *Neisseria* spp., termed the Correia repeats, may insert near sRNA genes and alter their functions [49]. This is similar to what is observed with QMITE1, especially those inserts that give rise to CbsR3 and CbsR13, two confirmed sRNAs harboring their own promoters upstream of the QMITE1 inserts and terminating within the confines of the insert itself (see Additional file 11) [30]. When taking the unambiguous reads associated with all QMITE1 loci into account, the TPMs associated with QMITE1 loci reach approximately 9342, or 0.93% of all transcripts expressed by *C. burnetii*. The fact that these promoter elements still exist after divergence of *C. burnetii* into separate strains speaks to the potential utility of the transcripts they produce, whether they: a) act *in trans* on mRNA target(s), b) affect expression of neighboring genes, or c) are actively translated to produce the high pI proteins listed in Additional file 5.

The truly unique aspect of QMITE inserts is the sRNAs they may produce wherever they insert into the genome. It has been shown that the Correia repeats of *N. meningitidis* give rise to transcripts that are produced at varying levels depending on the specific repeat in question [50]. Here, we confirm this notion by showing that a QMITE2 insert in the coding region of the lowly transcribed sRNA CbsR16 provides the − 10 promoter element for the sRNA (see Additional file 11). Additionally, this seems to be one, if not the only, QMITE2 insert that is transcribed with near-equivalence of the ambiguous and unambiguous TPM data (see Additional file 3). Additionally, although sRNAs arising from internal QMITE1 promoters have not been established, it is likely that transcripts are being produced by these inserts since many more ambiguous transcripts are associated with these loci than unambiguous transcripts (see Additional file 3). As seen in Additional file 1, these QMITE1 insertions also have identifiable promoters on both strands of DNA.

In general, ORFs that are affected by QMITE insertion events were the same between the two strains analyzed. One exception occurs in the Dugway strain’s *enhC* gene, which codes for a protein that is thought to inhibit release of peptide fragments during infection by *Legionella pneumophila*, *C. burnetii*’s closest pathogenic relative [51, 52]. The function of EnhC in *C. burnetii*’s pathogenicity has not yet been established, although recent studies have speculated that it may play a similar role to the *L. pneumophila* counterpart [53]. In the Dugway strain, *enhC* is extended due to an in-frame QMITE2 insertion at the 3′ end of the gene. Thus, the C-terminal 33 amino acids are presumably provided by the QMITE2 insertion, and the stop codon occurs immediately downstream. This same QMITE2 insert also exists in RSA 493, although an indel has resulted in a stop codon immediately preceding the element. It is unclear whether the C-terminal extension in Dugway affects EnhC function when compared to the altered protein product expressed by RSA 493. Conceivably, as the Dugway EnhC mRNA is transcribed, the highly stable stem structure conferred by QMITE2 could serve as a substrate for ribonuclease III processing. This could create an mRNA lacking a stop codon, which would, in turn, lead to ribosome stalling and eventual targeting of the nascent polypeptide for degradation [54]. Whether this process occurs as hypothesized is currently under investigation.

## Conclusions

A variety of TEs have been previously described in *C. burnetii*. Here, we have characterized two novel MITE families that exist as multiple copies in all annotated strains of *C. burnetii*. QMITE1 is of importance because its promoter elements could influence expression of nearby genes. QMITE2 is noteworthy due to unique DRs that could allow for identification of syntenic blocks and visualization of chromosomal rearrangements that have occurred between *C. burnetii* strains as they diverged. QMITE loci could also be used to identify chromosomal regions derived through HGT after the QMITE copies became inactive but prior to divergence of strains. The linkage conservation between QMITE1 and QMITE2 elements has helped us establish a timeline that suggests that these elements helped influence the evolution of *C. burnetii* on its path towards becoming an obligate pathogen by serving as sites for IS1111 transposition and inserting into and influencing annotated ORFs and sRNA genes. Finally, we have described the influence that QMITE insertions have had on CbsR3, CbsR13, and CbsR16 sRNA’s, the latter of which is produced from a promoter element within a QMITE2 insert.

## Additional files

Additional file 1: Extended-QMITE1 sequence for discontiguous megaBLAST searches. Predicted sigma-70 promoter elements for: Forward − 10 (red), Forward − 35 (red); Reverse − 10 (blue), Reverse − 35 (blue). (TIF 55 kb)

Additional file 2: Maximum likelihood phylogenetic tree of QMITE1 inserts. Node labels are indicated at the corresponding locations, and a branch length legend is shown at the bottom of the figure. (PDF 11 kb)

Additional file 3: QMITE-associated TPMs obtained by RNA-Seq from *C. burnetii* LCVs grown in infected Vero cells (*n* = 2 biological replicates). (TIF 30 kb)

Additional file 4: MUSTv2 search results indicating identified QMITE1 elements in the *C. burnetii* RSA 493 genome. Attributes of individual MITES are shown. (TIF 48 kb)

Additional file 5: MUSCLE alignment of RSA 493 DUF1658 proteins. (PDF 118 kb)

Additional file 6: Maximum likelihood phylogenetic tree of full-size QMITE2 inserts. Node labels are indicated at the corresponding locations, and a branch length legend is shown at the bottom of the figure. (PDF 9 kb)

Additional file 7: Maximum likelihood phylogenetic tree of small QMITE2 inserts. Node labels are indicated at the corresponding locations, and a branch length legend is shown at the bottom of the figure. (PDF 10 kb)

Additional file 8: MUSCLE alignment of transposon-associated QMITE2 inserts. (PDF 176 kb)

Additional file 9: Maximum likelihood phylogenetic tree of transposon-associated QMITE2 inserts. Node labels are indicated at the corresponding locations, and a branch length legend is shown at the bottom of the figure. (PDF 10 kb)

Additional file 10: Maximum likelihood phylogenetic tree of QMITE2 inserts found in alphaproteobacteria. Node labels are indicated at the corresponding locations, and a branch length legend is shown at the bottom of the figure. (PDF 18 kb)

Additional file 11: QMITE insertions in functional sRNAs of *C. burnetii*. (TIF 118 kb)

## Abbreviations

bp: Base pairs
DR: Direct repeat
IHF: Integration host factor
IS: Insertion sequence
MITE: Miniature inverted-repeat transposable element
nt: Nucleotide(s)
ORF: Open reading frame
pI: Isoelectric point
REP: Repetitive extragenic palindrome
sRNA: Small non-coding RNA
TE: Transposable element
TIR: Terminal inverted repeat
