# Supplementary figures and images for: Identification of novel MITEs (miniature inverted-repeat transposable elements) in Coxiella burnetii: implications for protein and small RNA evolution
Source: BMC Genomics. 2018 Apr 11;19:247. doi: 10.1186/s12864-018-4608-y (PMC5896051; doi:10.1186/s12864-018-4608-y)

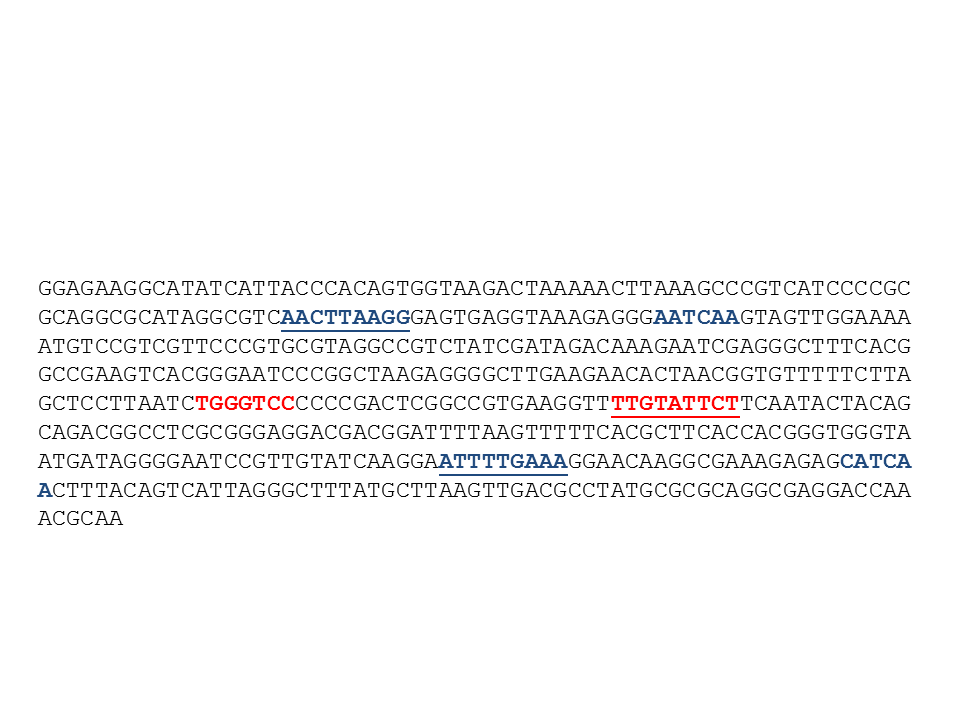

Supplement: Supplementary file 1 — Extended-QMITE1 sequence for discontiguous megaBLAST searches. Predicted sigma-70 promoter elements for: Forward − 10 (red), Forward − 35 (red); Reverse − 10 (blue), Reverse − 35 (blue). (TIF 55 kb) [file 12864_2018_4608_MOESM1_ESM.tif]

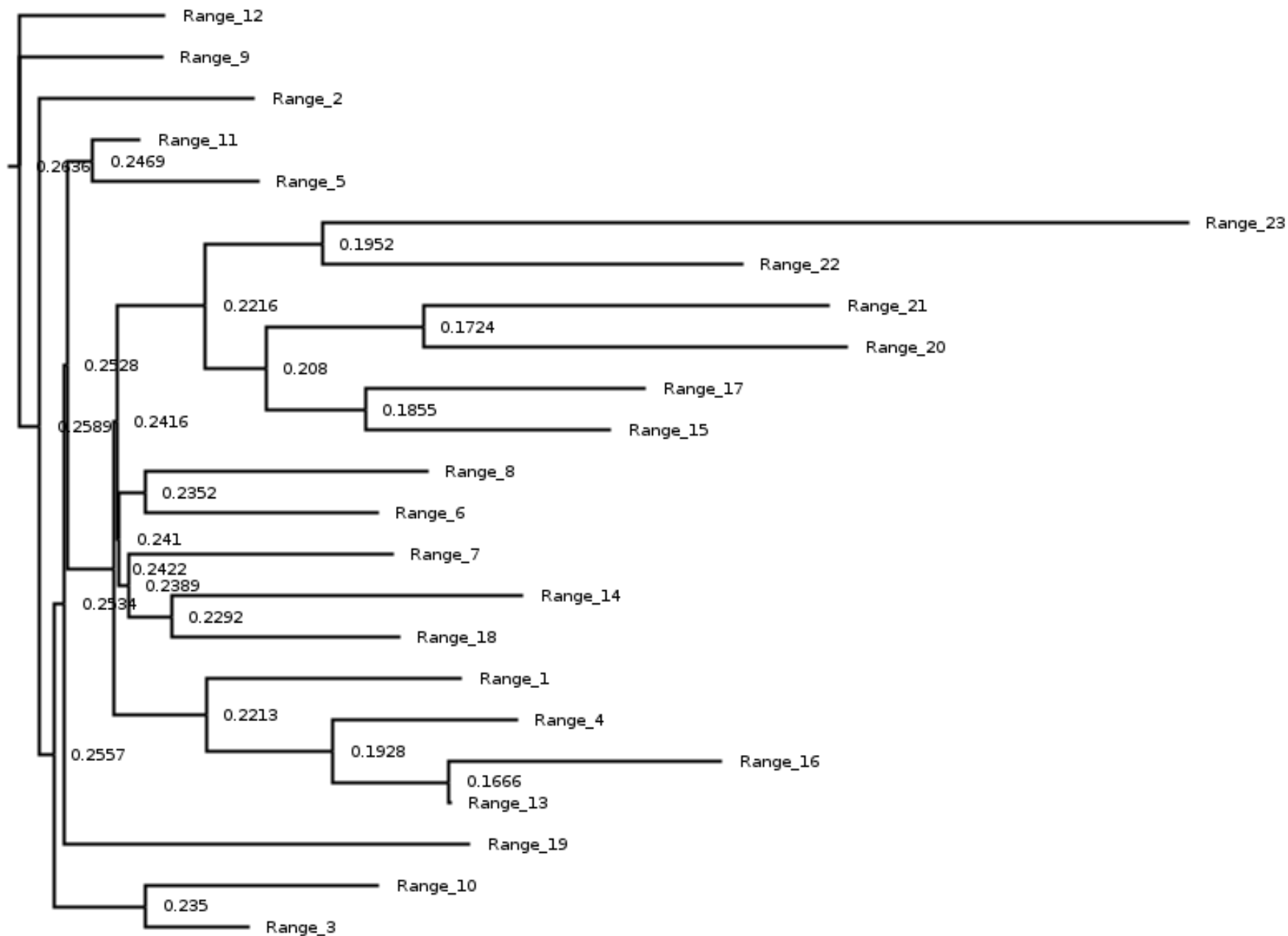

0.03

Supplement: Supplementary file 2 — Maximum likelihood phylogenetic tree of QMITE1 inserts. Node labels are indicated at the corresponding locations, and a branch length legend is shown at the bottom of the figure. (PDF 11 kb) [file 12864_2018_4608_MOESM2_ESM.pdf]

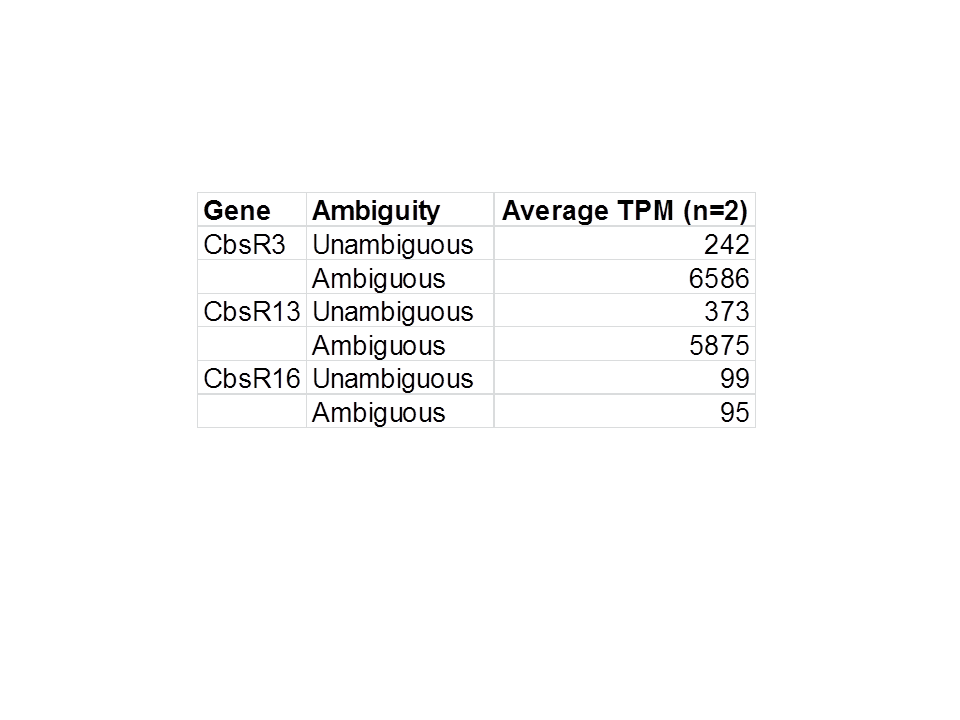

Supplement: Supplementary file 3 — QMITE-associated TPMs obtained by RNA-Seq from C. burnetii LCVs grown in infected Vero cells (n = 2 biological replicates). (TIF 30 kb) [file 12864_2018_4608_MOESM3_ESM.tif]

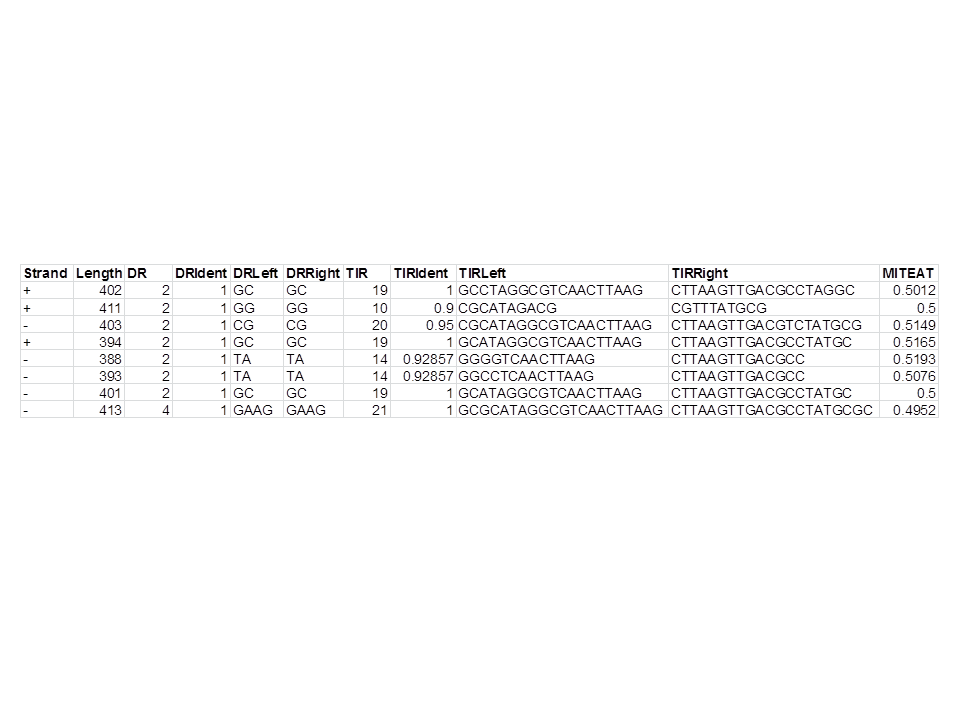

Supplement: Supplementary file 4 — MUSTv2 search results indicating identified QMITE1 elements in the C. burnetii RSA 493 genome. Attributes of individual MITES are shown. (TIF 48 kb) [file 12864_2018_4608_MOESM4_ESM.tif]

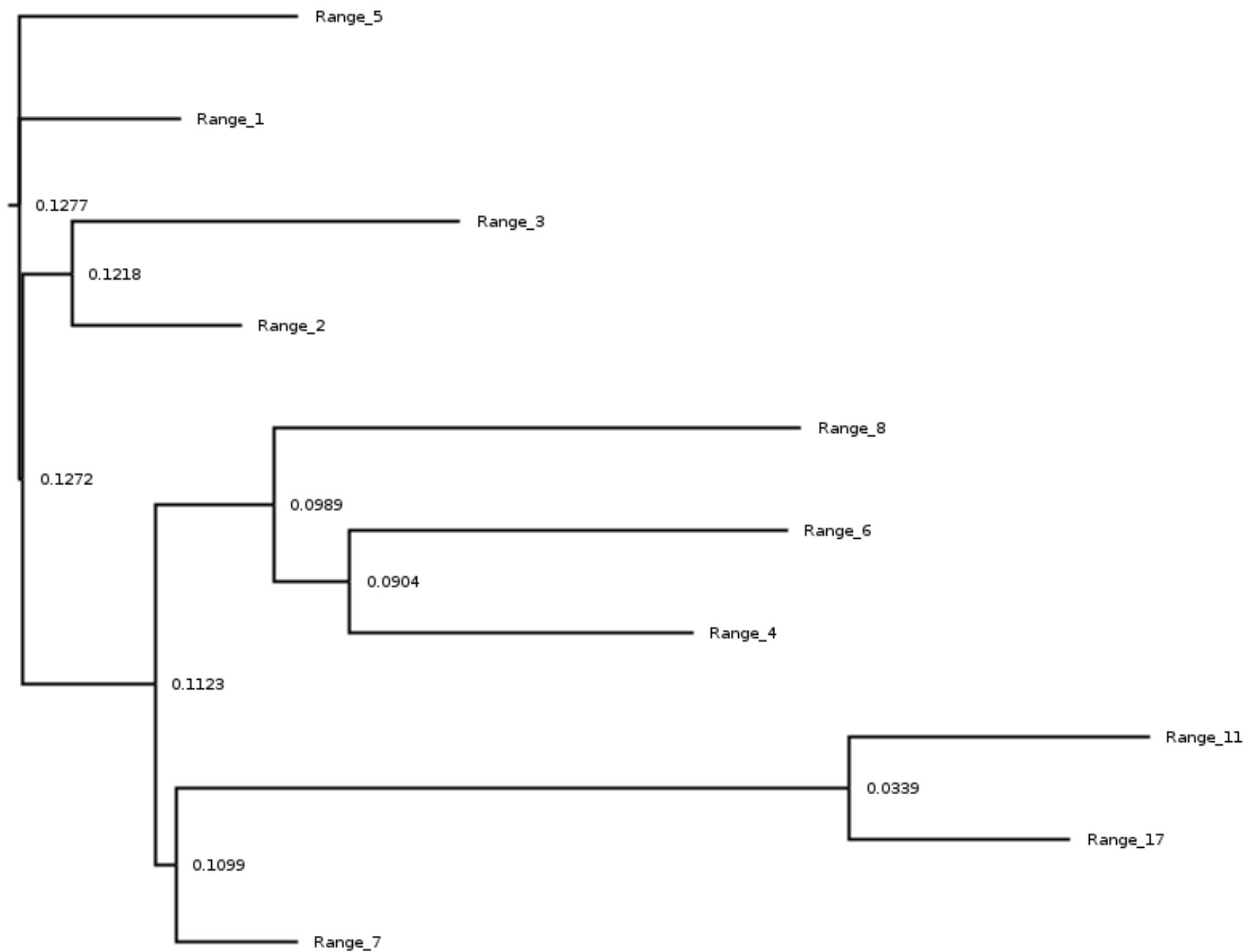

0.02

Supplement: Supplementary file 6 — Maximum likelihood phylogenetic tree of full-size QMITE2 inserts. Node labels are indicated at the corresponding locations, and a branch length legend is shown at the bottom of the figure. (PDF 9 kb) [file 12864_2018_4608_MOESM6_ESM.pdf]

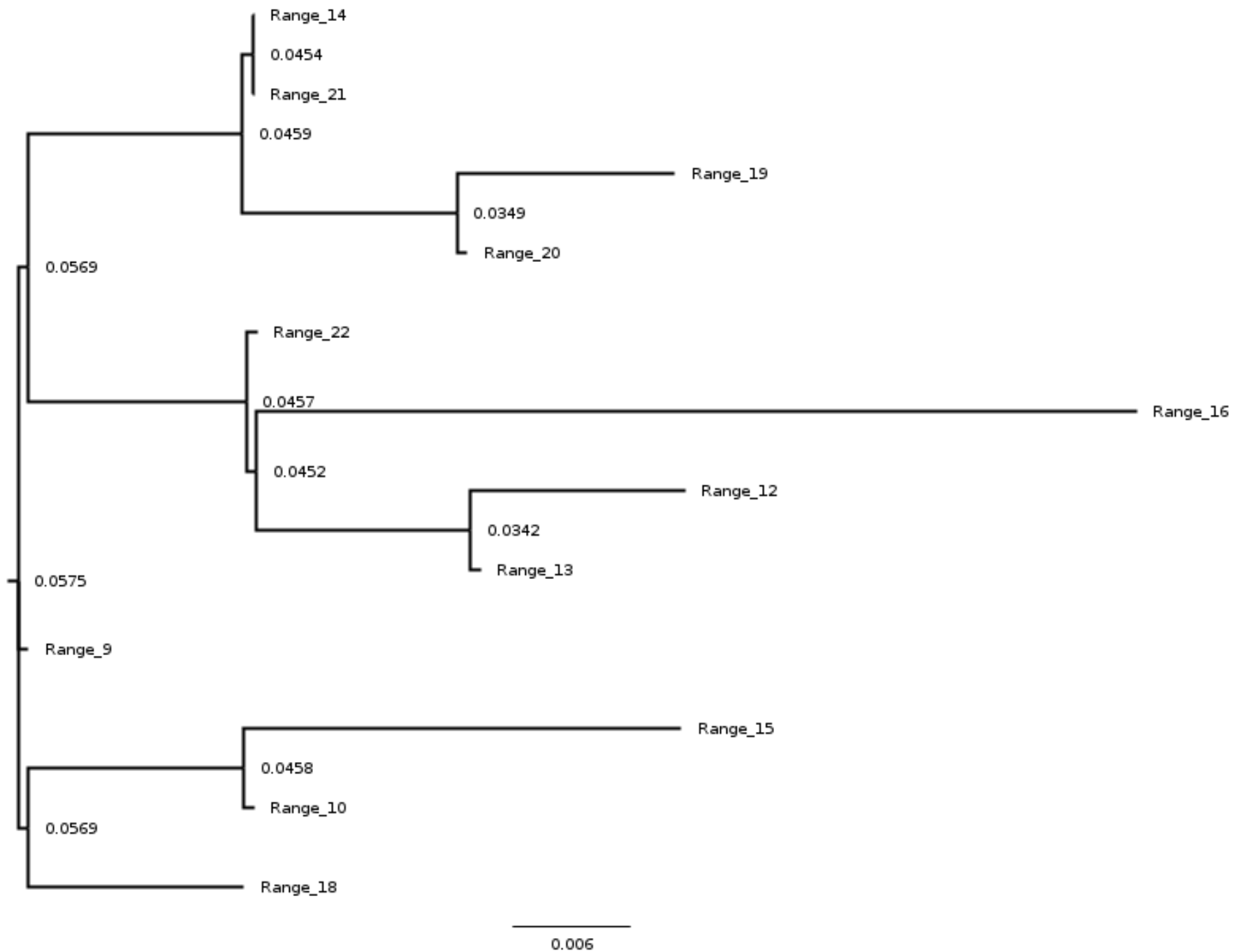

Supplement: Supplementary file 7 — Maximum likelihood phylogenetic tree of small QMITE2 inserts. Node labels are indicated at the corresponding locations, and a branch length legend is shown at the bottom of the figure. (PDF 10 kb) [file 12864_2018_4608_MOESM7_ESM.pdf]

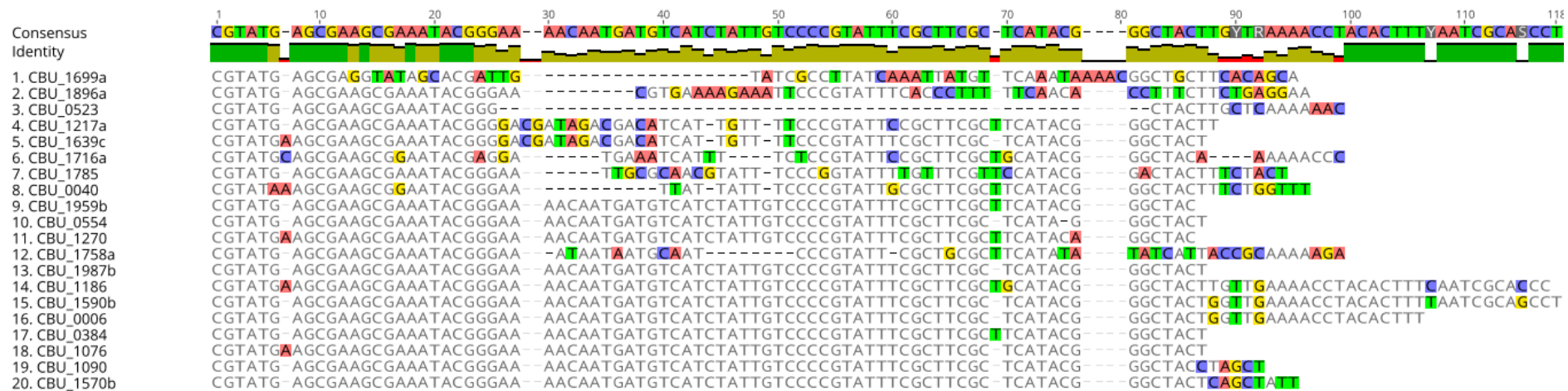

Supplement: Supplementary file 8 — MUSCLE alignment of transposon-associated QMITE2 inserts. (PDF 176 kb) [file 12864_2018_4608_MOESM8_ESM.pdf]

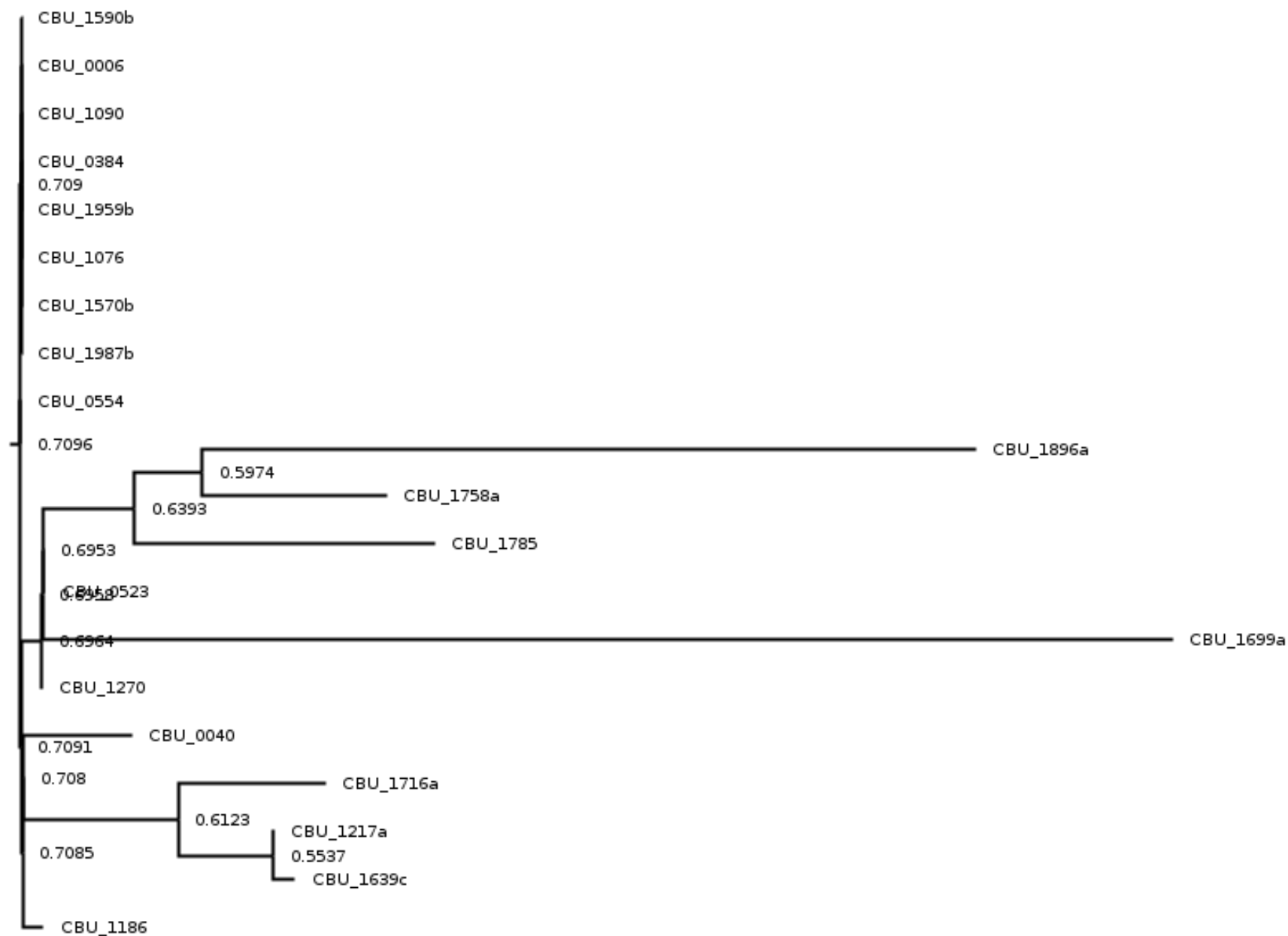

0.08

Supplement: Supplementary file 9 — Maximum likelihood phylogenetic tree of transposon-associated QMITE2 inserts. Node labels are indicated at the corresponding locations, and a branch length legend is shown at the bottom of the figure. (PDF 10 kb) [file 12864_2018_4608_MOESM9_ESM.pdf]

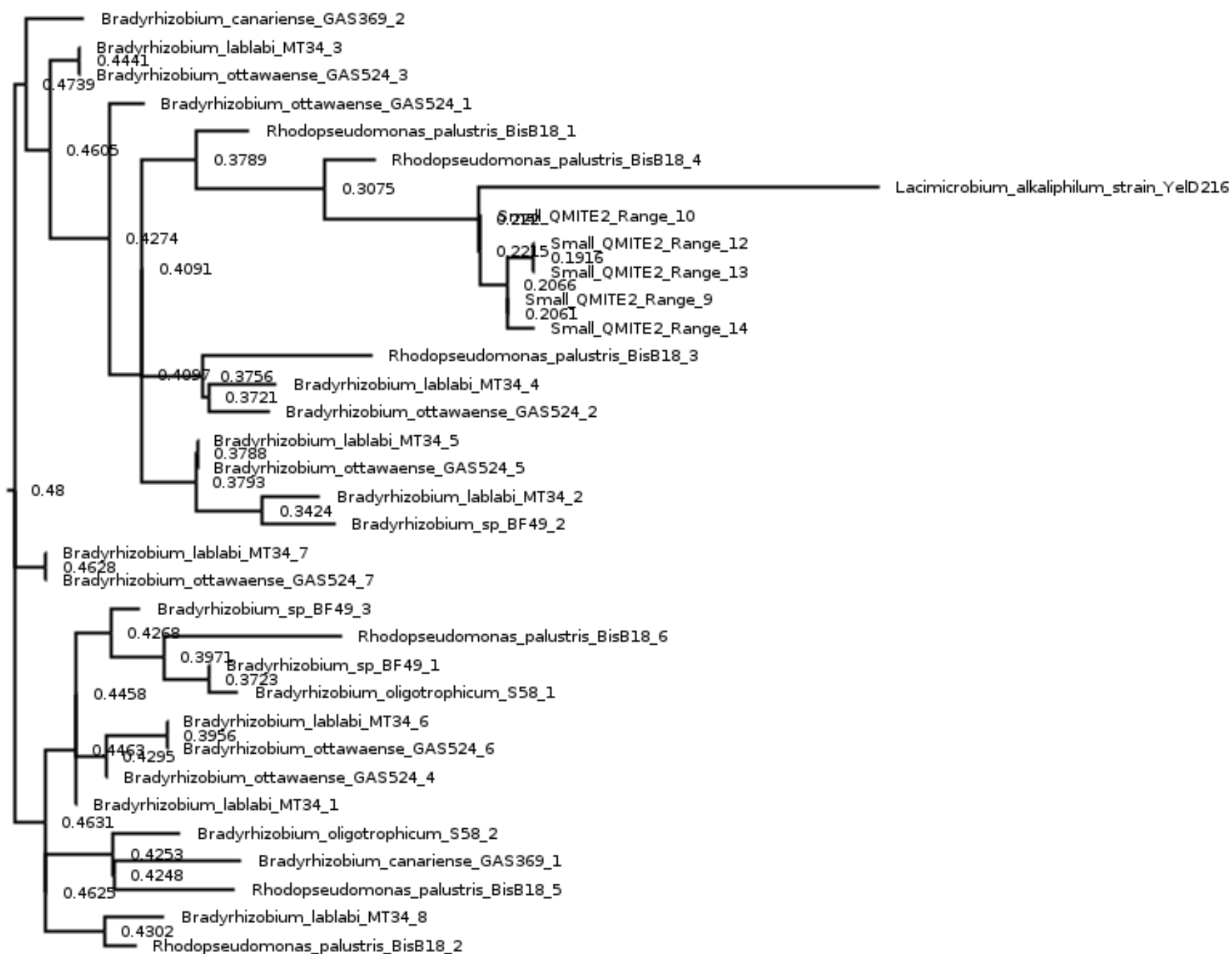

0.05

Supplement: Supplementary file 10 — Maximum likelihood phylogenetic tree of QMITE2 inserts found in alphaproteobacteria. Node labels are indicated at the corresponding locations, and a branch length legend is shown at the bottom of the figure. (PDF 18 kb) [file 12864_2018_4608_MOESM10_ESM.pdf]

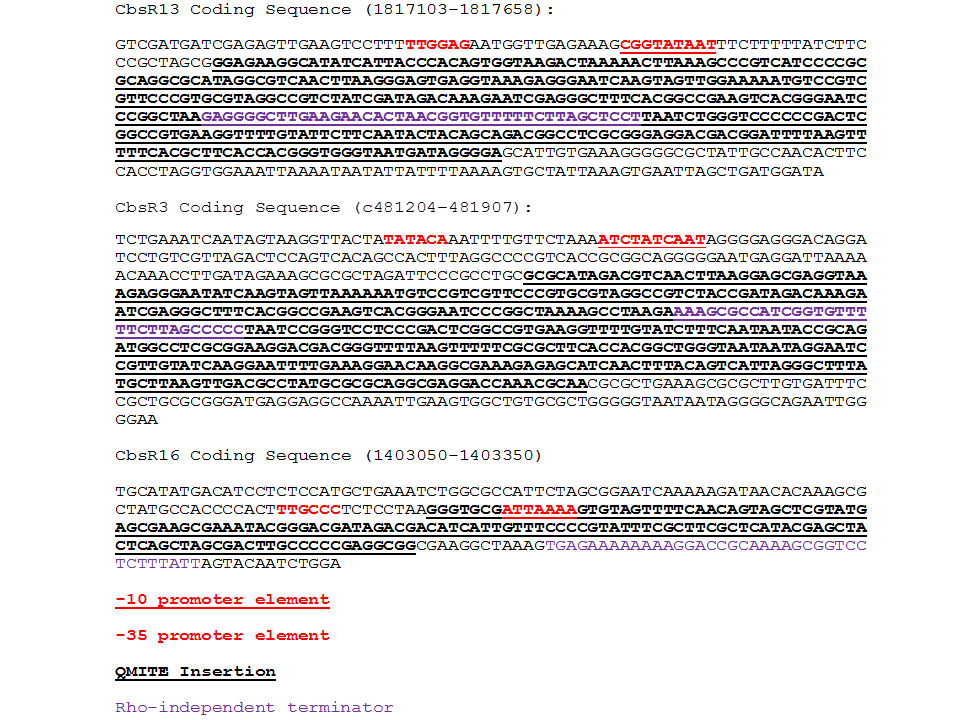

Supplement: Supplementary file 11 — QMITE insertions in functional sRNAs of C. burnetii. (TIF 118 kb) [file 12864_2018_4608_MOESM11_ESM.tif]
