# Supplementary material for: Identification of novel MITEs (miniature inverted-repeat transposable elements) in Coxiella burnetii: implications for protein and small RNA evolution
Source: BMC Genomics. 2018 Apr 11;19:247. doi: 10.1186/s12864-018-4608-y (PMC5896051; doi:10.1186/s12864-018-4608-y)
Supplement: Supplementary file 5 — MUSCLE alignment of RSA 493 DUF1658 proteins. (PDF 118 kb) [file 12864_2018_4608_MOESM5_ESM.pdf]

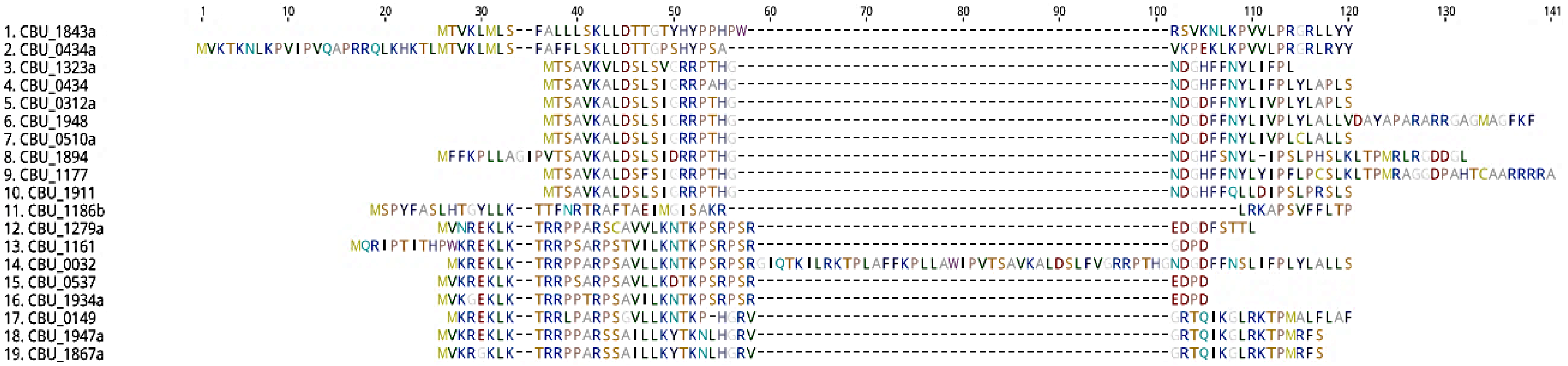

1 10 20 30 40 50 60 70 80 90 100 110 120 130 141
1. CBU_1843a MTVKLMLS--FALLLSKLLDTTGTYHYPPHPW--------------------------------------------RSVKNLKPVVLPRGRLLYY
2. CBU_0434a MVKTKNLKPVIPVQAPRRQLKHKTLMTVKLMLS--FAFFLSKLLDTTGPSHYPSA----------------------------------------------VKPEKLKPVVLPRGRLRYY
3. CBU_1323a MTSAVKVLDSLSVGRRPTHG----------------------------------------------NDGHFFNYLIFPL
4. CBU_0434 MTSAVKALDSLSIGRRPAHG----------------------------------------------NDGHFFNYLIFPLYLAPLS
5. CBU_0312a MTSAVKALDSLSIGRRPTHG----------------------------------------------NDGDFFNYLIVPLYLAPLS
6. CBU_1948 MTSAVKALDSLSIGRRPTHG----------------------------------------------NDGDFFNYLIVPLYLALLVDAYAPARARRGAGMAGFKF
7. CBU_0510a MTSAVKALDSLSIGRRPTHG----------------------------------------------NDGDFFNYLIVPLCLALLS
8. CBU_1894 MFFKPLLAGIPVTSAVKALDSLSIDRRPTHG----------------------------------------------NDGHFSNYL-IPSLPHSLKLTPMRLRGDDGL
9. CBU_1177 MTSAVKALDSFSIGRRPTHG----------------------------------------------NDGHFFNYLYIPFLPCSLKLTPMRAGGDPAHTCAARRRRA
10. CBU_1911 MTSAVKALDSLSIGRRPTHG----------------------------------------------NDGHFFQLLDIPSLPRSLS
11. CBU_1186b MSPYFASLHTGYLLK--TTFNRTRAFTAEIMGISAKR-----------------------------------------------------LRKAPSVFFLTP
12. CBU_1279a MVNREKLK--TRRPPARSCAVVLKNTKPSRPSR-------------------------------------------EDGDFSTTL
13. CBU_1161 MQRIPTITHPWKREKLK--TRRPSARPSTVILKNTKPSRPSR-------------------------------------------GDPD
14. CBU_0032 MKREKLK--TRRPPARPSAVLLKNTKPSRPSRGIQTKILRKTPLAFFKPLLAWIPVTSAVKALDSLFVGRRPTHGNDGDFFNSLIFPLYLALLS
15. CBU_0537 MVKREKLK--TRRPSARPSAVLLKDTKPSRPSR-------------------------------------------EDPD
16. CBU_1934a MVKGEKLK--TRRPPTRPSAVILKNTKPSRPSR-------------------------------------------EDPD
17. CBU_0149 MKREKLK--TRRLPARPSGVLLKNTKP-HGRV-------------------------------------------GRTQIKGLRKTPMALFLAF
18. CBU_1947a MVKREKLK--TRRPPARSSAILLKYTKNLHGRV-------------------------------------------GRTQIKGLRKTPMRFS
19. CBU_1867a MVKRGKLK--TRRPPARSSAILLKYTKNLHGRV-------------------------------------------GRTQIKGLRKTPMRFS
